# METTL3-mediated m6A modification of circCDKAL1 regulates macrophage M1 polarization and nasal epithelial cell barrier function in allergic rhinitis through IGF2BP2/JARID2/HMGB1 axis

**DOI:** 10.1038/s41420-025-02710-7

**Published:** 2025-08-29

**Authors:** Jiabin Zhan, Dan Luo, Yunlong Fu, Yu Zhou, Rui Li, Xin Wei

**Affiliations:** Department of Otorhinolaryngology Head and Neck Surgery, Hainan General Hospital (Hainan Affiliated Hospital of Hainan Medical University), Haikou City, Hainan Province PR China

**Keywords:** Respiratory tract diseases, Medical research

## Abstract

Allergic rhinitis (AR) is a chronic inflammatory disease that significantly impairs patients’ quality of life, with CircCDKAL1 showing abnormal upregulation in AR patients, though its functional mechanisms remain unclear. In this study, we confirmed the identity of circCDKAL1 and its subcellular localization. Our findings revealed that circCDKAL1 expression was enhanced in samples of AR patients, AR mice and OVA-induced HNEpCs. Besides, circCDKAL1 silencing resulted in improvement of nasal mucosal epithelial barrier function/epithelial cell adhesion and promotion of macrophage M2 polarization in AR. Mechanistically, we discovered that circCDKAL1 was modified by m6A, which was mediated by METTL3. YTHDC1 promoted cytoplasmic output of m6A-modified circCDKAL1. In addition, circCDKAL1 destabilized JARID2 mRNA through interacting with IGF2BP2. Moreover, circCDKAL1 or HMGB1 silencing attenuated JARID2 silencing-mediated damages of epithelial cell adhesion and promotion of macrophage M1 polarization in OVA-induced HNEpCs. In conclusion, METTL3-mediated m6A modification of circCDKAL1, which was transferred from the nucleus to the cytoplasm by YTHDC1, promoted macrophage M1 polarization and impaired nasal mucosal epithelial barrier function/epithelial cell adhesion in AR through interacting with IGF2BP2 and regulating JARID2/HMGB1 axis.

## Introduction

Allergic rhinitis (AR) is an allergic disease, caused by a specific organism after contact with allergens and mediated mainly by immunoglobulin E (IgE) and a variety of inflammatory factors. The global incidence of AR is increasing every year, reaching up to 50% in some developed countries [1]. Generally speaking, AR is difficult to cause death directly, but its accompanying symptoms such as sneezing, runny nose, itching, stuffiness, and bleeding bring trouble to patients and lower their quality of life [2, 3]. Current therapy for AR mainly includes avoidance of allergens, medication and immunotherapy. However, due to the complex and unclear pathogenesis of AR, current treatments still have significant limitations [4]. Therefore, deeply exploring the molecular regulatory mechanisms of AR will help to understand underlying pathogenesis of AR and seek methods for its treatment.

Circular RNAs (circRNAs), important components of non-coding RNAs (ncRNAs), have a closed loop structure and do not have a 5 ‘terminal cap or a 3’ terminal poly (A) tail, which play crucial roles in various diseases [5]. For example, some circRNAs, such as, circHIPK3 and circ_0067835, were implicated in AR progression [6, 7]. Employing high-throughput sequencing (HTS), a previous study revealed that numerous circRNAs were abnormally downregulated or upregulated in nasal mucosa tissues from AR patients [8]. Based on this study [8], upregulated expression of circCDKAL1 attracted our attention. Currently, circCDKAL1 has rarely been reported in diseases, so we desired to explore why it was abnormally elevated in AR patients and how it affects AR progression. It is well-known that RNA N6-methyladenosine (m6A) modification is a common and important epigenetic modification mode in post-transcriptional regulation, which modification is widely found in eukaryotes mRNAs and ncRNAs, and can regulate alternative splicing, mRNA exucleation transport, mRNA stability and mRNA translation [9]. These m6A modification-mediated functions have been reported to depend on conformational changes within local RNA structures or the recruitment of specific m6A reader proteins [9, 10]. Rmbase predicted that circCDKAL1 had abundant m6A methylation modification sites. In addition, we predicted that there harbored potential binding site between the m6A methyltransferase METTL3 and circCDKAL1. Similarly, we also predicted that circCDKAL1 had binding site on m6A recognition protein YTHDC1. Thus, we assumed that the functions and mechanism of circCDKAL1 in AR may be related to m6A modification, which will be verified by a series of experiments.

Previous reports have suggested that circRNAs may be involved in the onset and development of diseases through a variety of molecular regulatory mechanisms such as miRNA sponge, alternative splicing and RNA-binding protein (RBP) interactions [11, 12]. An increasing number of studies have demonstrated that circRNAs bind to RBPs to enhance or reduce the mRNA stability of targeted genes in diseases [13]. Insulin-like growth factor 2 mRNA-binding protein 2 (IGF2BP2), a common RBP, was broadly reported to interact with circRNAs [14]. In addition, Hyo Jin Kim and co-workers suggested that jumonji and AT-rich interaction domain containing 2 (JARID2) was implicated in airway inflammation [15], indicating JARID2 might play a role in AR progression. StarBase predicted that there were binding sites among JARID2, circCDKAL1 and IGF2BP2. Therefore, we speculated that circCDKAL1 may be involved in the pathological status of AR through interacting with RBP binding.

Based on the above-mentioned evidence, we put forward the hypothesis that YTHDC1 promotes cytoplasmic output of circCDKAL1 depending on an m6A modification, which was mediated by METTL3, and circCDKAL1 suppressed JARID2 mRNA stability to inhibit JARID2 expression through interacting with IGF2BP2, thereby enhancing high-mobility group box-1 protein 1 (HMGB1) expression to disrupt nasal epithelial cell adhesion and induced macrophage M1 polarization in AR.

## Results

### Characterization of circCDKAL1 was identified and circCDKAL1 expression was abnormally upregulated in AR

**Fig. 1:**
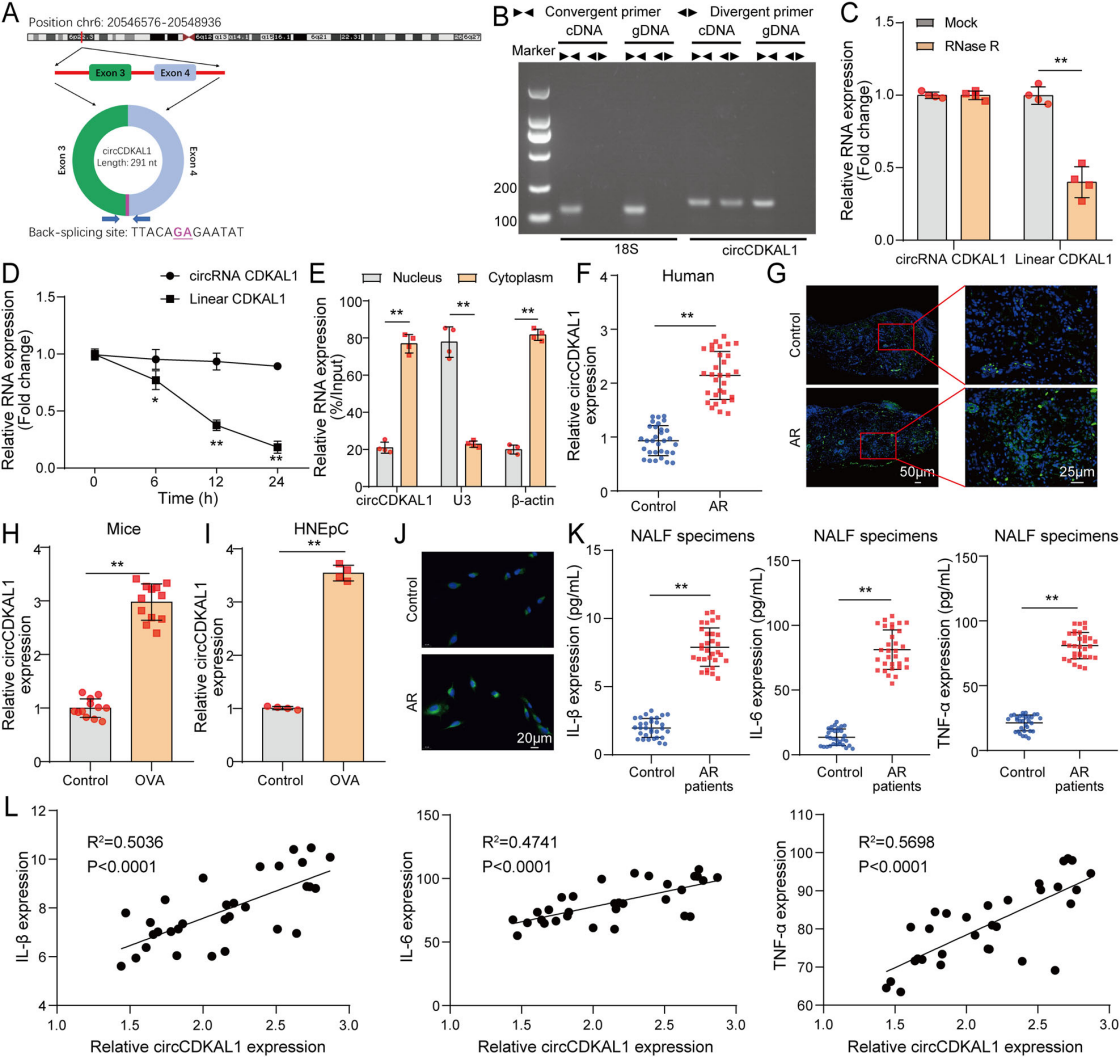
Characterization of circCDKAL1 was identified and circCDKAL1 expression was abnormally upregulated in AR. **A** The genomic loci and validation of circCDKAL1 via Sanger sequencing. **B** Divergent and convergent primers were designed and 18S and circCDKAL1 were amplified and detected by gel electrophoresis in gDNA and cDNA samples. *n* = 4. **C** CircCDKAL1 and linear CDKAL1 expression were measured by RT-qPCR in HNEpCs upon RNase R treatment. *n* = 4. **D** CircCDKAL1 and linear CDKAL1 expression were determined by RT-qPCR in HNEpCs upon actinomycin D treatment at various time points. *n* = 4. **E** Cellular localization of circCDKAL1 in HNEpCs was determined using nuclear/cytoplasmic fractionation experiment. *n* = 4. **F** CircCDKAL1 expression in NALF of AR patients was detected using RT-qPCR. *n* = 12. (**G**) CircCDKAL1 in nasal mucosa epithelium and submucosa of AR patients was examined by FISH assay. *n* = 12. **H**, **I** CircCDKAL1 expression in nasal mucosa tissues of AR mice (*n* = 12) and OVA-induced HNEpCs (*n* = 3) was measured by RT-qPCR. **J** The distribution and expression of circCDKAL1 in OVA-induced HNEpCs were evaluated by FISH assay. *n* = 4. **K** The levels of inflammatory cytokines in NALF of AR patients were detected using ELISA. *n* = 12. **L** The relationship between circCDKAL1 and IL-1β/IL-6/TNF-α expression in NALF of AR patients was analyzed using Pearson correlation analysis. *P < 0.05, **P < 0.01.

As presented in Fig. 1A, depending on circbase (http://www.circbase.org), we learned that circCDKAL1 was located at chr 6:20546576-20548936 and was circularized by exon 3-4 of CDKAL1 with a length of 291nt. Meanwhile, the back-spliced junction of circCDKAL1 was validated by Sanger sequencing. In addition, agarose gel electrophoresis results exhibited that convergent primer could amplify products in gDNA and cDNA, while divergent primer could only amplify circCDKAL1 in cDNA (Fig. 1B). Subsequently, RNase R digestion assay displayed that circCDKAL1 resisted the digestion by RNase R, while linear CDKAL1 significantly degraded in HNEpCs (Fig. 1C). After HNEpCs were treated with actinomycin D, we observed that the half-life of circCDKAL1 RNA was longer than that of linear CDKAL1 RNA (Fig. 1D). Nuclear/cytoplasmic fractionation experiment showed that circCDKAL1 was distributed in both cytoplasm and nucleus of HNEpCs and the results revealed that circCDKAL1 was mainly localized in the cytoplasm (Fig. 1E). These above-mentioned results indicated that circCDKAL1 was a stable circRNA expressed in HNEpCs and located in the cytoplasm.

A study published in 2021 pointed out that circCDKAL1 expression was upregulated in AR patients through HTS [8]. Here, we detected circCDKAL1 expression in NALF of patients with AR and upregulation of circCDKAL1 expression was observed in NALF samples (Fig. 1F). FISH assay exhibited that the fluorescence intensity of circCDKAL1 in the nasal mucosa was significantly increased in AR patients (Fig. 1G). In addition, in OVA-induced mice, OVA induction evidently enhanced circCDKAL1 expression in nasal mucosa tissues (Fig. 1H). Similarly, in OVA-induced HNEpCs, circCDKAL1 expression also was apparently higher than HNEpCs without OVA induction (Fig. 1I). FISH assay revealed that circCDKAL1 was mainly localized in cytoplasm of HNEpCs, which distribution in cytoplasm was signally increased in OVA-induced HNEpCs (Fig. 1J). As for the levels of inflammatory cytokines including IL-1β, IL-6 and TNF-α in NALF of AR patients, we observed elevating levels of inflammatory cytokines in AR samples (Fig. 1K). Furthermore, Pearson correlation analysis further disclosed that circCDKAL1 expression was positively related to IL-1β, IL-6 and TNF-α expression in NALF of AR patients (Fig. 1L). In total, circCDKAL1 expression was abnormally elevated in AR patients, OVA-induced mice and HNEpCs.

### CircCDKAL1 knockdown improved nasal mucosal injury and alleviated proinflammatory mucosal microenvironment in AR mice

**Fig. 2:**
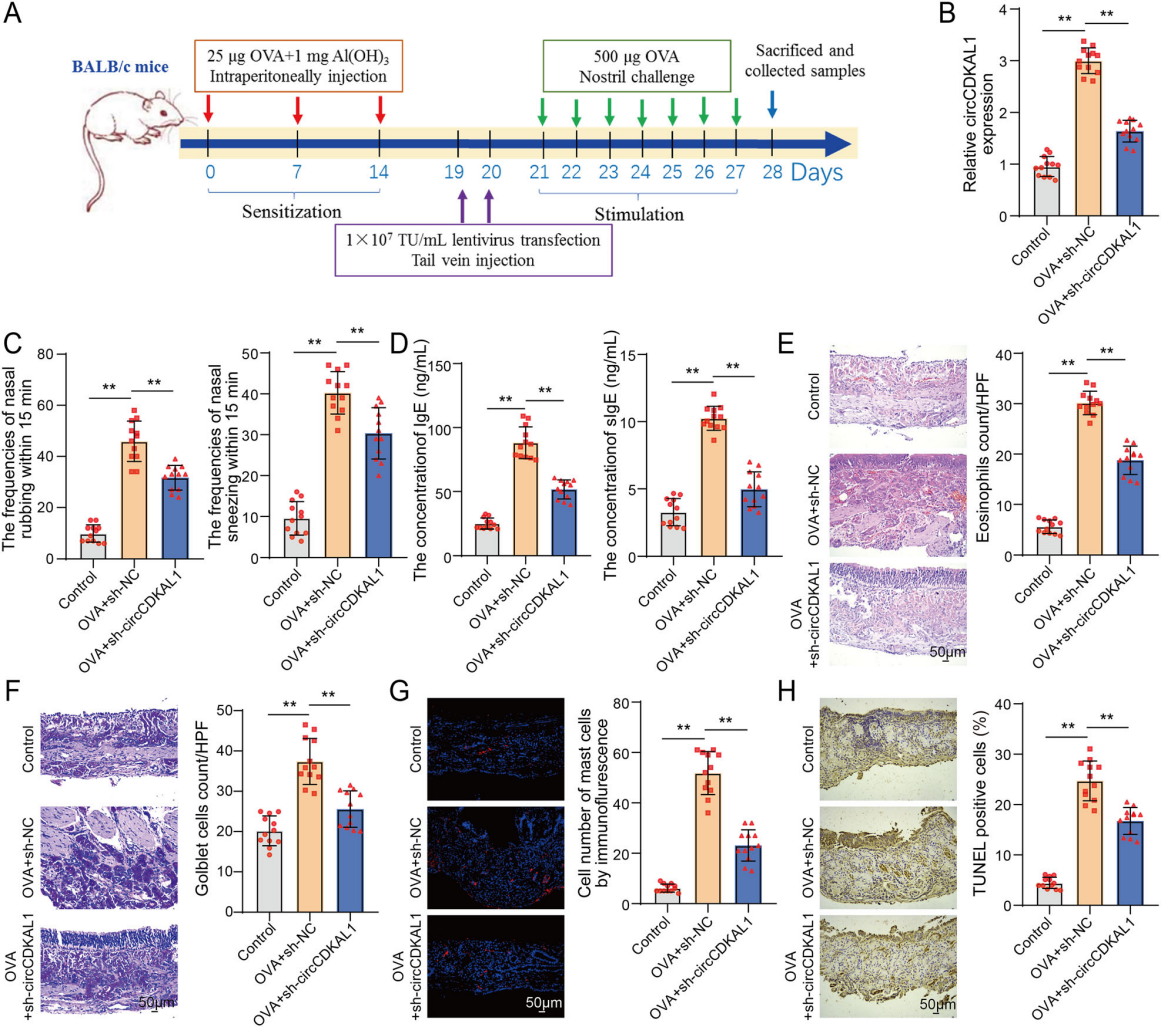
CircCDKAL1 knockdown improved nasal mucosal injury and alleviated proinflammatory mucosal microenvironment in AR mice. **A** The flowchart of constructing AR mice model and intervening plan. **B** circCDKAL1 expression was detected by RT-qPCR. *n* = 12. **C** Frequencies of sneezing and nasal rubbing were observed and recorded. *n* = 12. **D** The concentrations of IgE and sIgE in serum of mice were determined using ELISA. *n* = 12. **E** Histopathological changes of nasal mucosa of mice were evaluated using HE staining. *n* = 12. **F** The goblet cells of nasal mucosa of mice were observed by PAS staining. *n* = 12. **G** Mast cell infiltration of the nasal mucosa of mice was detected by IF assay. *n* = 12. **H** Cell apoptosis of nasal mucosa tissues of mice was measured using TUNEL. *n* = 12. **P < 0.01.

To probe the role of circCDKAL1 in AR, firstly, an AR model of mice was established and lv-sh-circCDKAL1 was injected into AR mice. The detailed processes of AR mice and lv-sh-circCDKAL1 injection were shown in Fig. 2A. In detail, mice received 25 μg OVA and 1 mg aluminum hydroxide gel at 0, 7, and 14 d through intraperitoneal injection and were challenged with 500 μg OVA from day 21 to day 27 through intranasal exposure. At 19 d, lv-sh-circCDKAL1 was injected into AR mice through tail vein. At 28 d, the mice were sacrificed and relevant samples were collected. According to the analysis in Fig. 2B, lv-sh-circCDKAL1 injection notably suppressed OVA-induced upregulation of circCDKAL1 expression. Besides, after the OVA intranasal challenge, we observed that OVA induction observably increased frequencies of sneezing and nasal rubbing within 15 min, whereas these manifestations were abolished by circCDKAL1 knockdown (Fig. 2C). The concentrations of IgE and sIgE in the serum of mice were elevated by OVA induction, while circCDKAL1 knockdown reversed OVA-induced concentrations of IgE and sIgE (Fig. 2D). HE staining displayed that epithelial thickness and eosinophilic infiltration in nasal mucosa were signally increased by OVA induction, which were reversed by circCDKAL1 knockdown (Fig. 2E). PAS staining indicated that the goblet cells of the nasal mucosa epithelium, which were responsible for mucus secretion, were increased by OVA induction, however, circCDKAL1 knockdown decreased goblet cells (Fig. 2F). Figure 2G discovered that OVA induction resulted in elevated mast cell infiltration of the nasal mucosa, which was compromised by circCDKAL1 knockdown. TUNEL assay found that OVA induction could promote cell apoptosis of nasal mucosa tissues, and circCDKAL1 knockdown attenuated OVA-induced cell apoptosis (Fig. 2H). Collectively, circCDKAL1 silencing alleviated histological injury and inhibited inflammatory infiltration of nasal mucosa in AR mice.

### CircCDKAL1 silencing protected the epithelial cell barrier function of nasal mucosa and induced macrophage M2 polarization in OVA-induced mice and HNEpCs

**Fig. 3:**
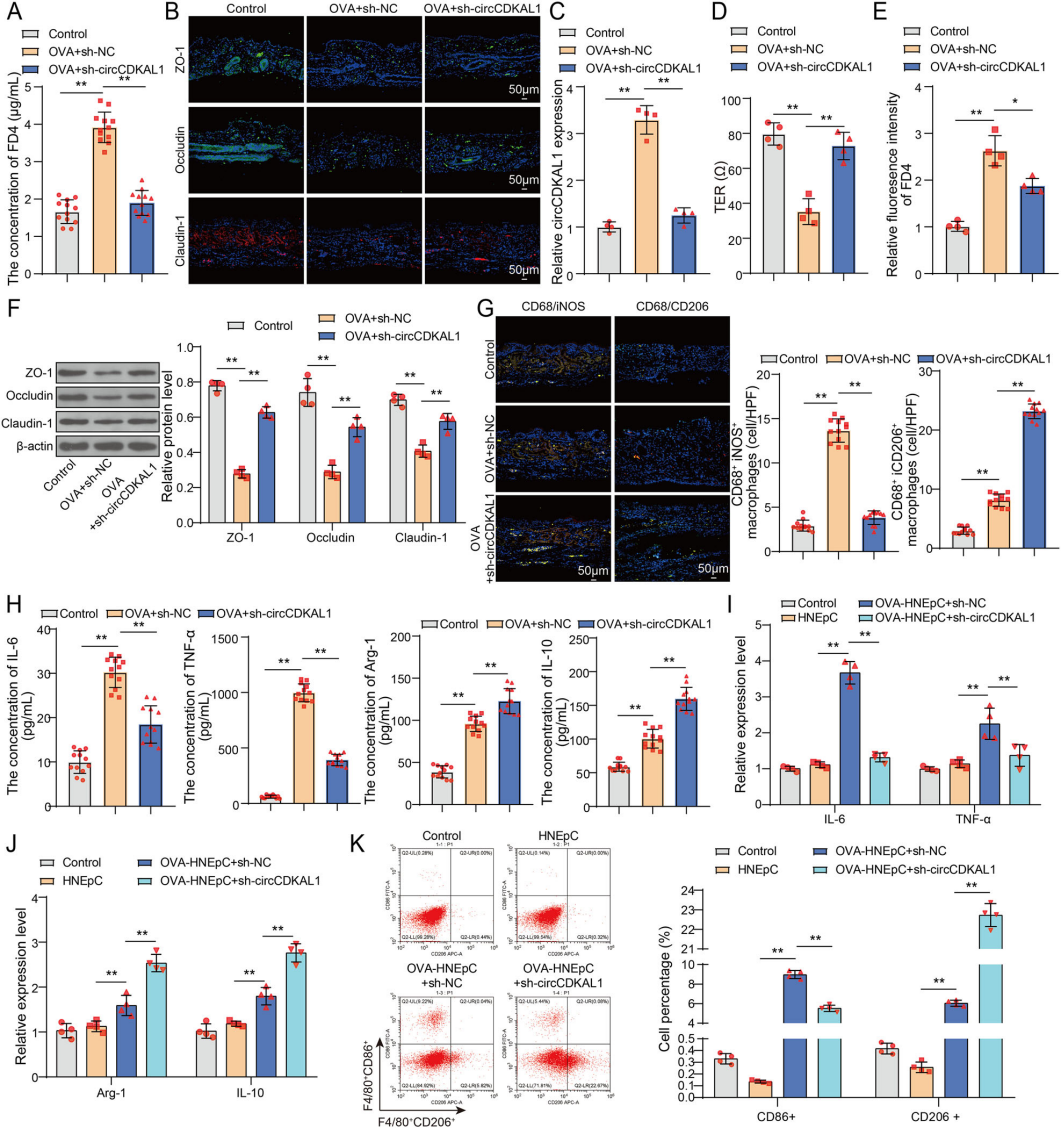
CircCDKAL1 silencing protected the epithelial cell barrier function of nasal mucosa and induced macrophage M2 polarizations in OVA-induced mice and HNEpCs. **A** FD4 levels in mice were evaluated using CLARIOstar. *n* = 12. **B** ZO-1, Occludin and Claudin-1 expression in nasal mucosa tissues of mice was determined using IF assay. *n* = 12. **C** circCDKAL1 expression in sh-NC- or sh-circCDKAL1-transfected HNEpCs upon OVA treatment was evaluated using RT-qPCR. *n* = 4. **D** TER was examined using EVOM/EndOhm system. *n* = 4. **E** FD4 levels in HNEpCs were evaluated using CLARIOstar. *n* = 4. **F** ZO-1, Occludin and Claudin-1 expression in HNEpCs was detected using western blot. *n* = 4. **G** The co-localization of CD68/iNOS as well as CD68/CD206 in nasal mucosa of AR mice was measured using double immunofluorescence staining. *n* = 12. **H** The levels of IL-6, TNF-α, Arg-1and IL-10 in serum of AR mice were determined by ELISA. *n* = 12. HNEpCs were subjected to sh-NC or sh-circCDKAL1 transfection and followed by OVA treatment, which were co-cultured with MH-S cells for 24 h. **I**, **J** The expression of IL-6, TNF-α, Arg-1and IL-10 in MH-S cells were examined by RT-qPCR. *n* = 4. **K** CD86^+^ and CD206^+^ positive macrophages were detected using flow cytometry. *n* = 4. *P < 0.05, **P < 0.01.

Subsequently, the epithelial barrier integrity was evaluated using FD4 permeability and detecting some indicators including ZO-1, Occludin and Claudin-1 in experimental mice. As presented in Fig. 3A, the level of FD4 in serum was elevated in AR mice, but circCDKAL1 silencing declined FD4 level. IF assay indicated that OVA induction led to decreased ZO-1, Occludin and Claudin-1 expression in nasal mucosa tissues, which was reversed by circCDKAL1 knockdown (Fig. 3B). Additionally, HNEpCs were subjected to sh-NC or sh-circCDKAL1 transfection and then followed by OVA treatment. OVA-induced increase of circCDKAL1 expression in HNEpCs was offset by sh-circCDKAL1 transfection (Fig. 3C). For evaluation of epithelial barrier integrity, TER was lower in OVA-induced HNEpCs than HNEpCs without OVA treatment, suggesting OVA induction damaged the epithelial barrier. As expected, circCDKAL1 silencing partially restored OVA-mediated lower TER (Fig. 3D). Consistent with the results in AR mice, FD4 level was increased in OVA-induced HNEpCs, which was reversed by circCDKAL1 silencing (Fig. 3E). Additionally, OVA-induced lower ZO-1, Occludin and Claudin-1 expression was abolished by circCDKAL1 silencing (Fig. 3F). Taken together, circCDKAL1 silencing reinforced the epithelial barrier integrity in OVA-induced mice and HNEpCs.

It was reported that macrophage polarization of M1 and M2 was closely related to AR progression [16]. Here, double immunofluorescence staining revealed that OVA induction increased the colocalization of CD68/iNOS as well as CD68/CD206 in nasal mucosa of mice, however, circCDKAL1 silencing attenuated OVA-mediated increasing colocalization of CD68/iNOS and further reinforced OVA-mediated increasing colocalization of CD68/CD206 (Fig. 3G). Moreover, the concentrations of IL-6 and TNF-α (markers of M1 macrophages polarization and the concentrations of Arg-1and IL-10 (markers of M2 macrophages polarization) were elevated in serum and NALF of OVA-induced mice, but circCDKAL1 silencing suppressed the concentrations of IL-6 and TNF-α and enhanced the concentrations of Arg-1 and IL-10 in OVA-induced mice (Fig. 3H and Supplementary Figure 1A). At the cellular level, HNEpCs were subjected to sh-NC or sh-circCDKAL1 transfection and followed by OVA treatment. Then, HNEpCs were co-cultured with MH-S cells for 24 h. The levels of IL-6, TNF-α, Arg-1 and IL-10 in MH-S cells were elevated by OVA treatment, but circCDKAL1 silencing declined OVA-induced IL-6 and TNF-α levels and further promoted OVA-induced Arg-1 and IL-10 levels (Fig. 3I, J). The results of flow cytometry exhibited that OVA induction resulted in increased CD86^+^ and CD206^+^ positive macrophages, while OVA-induced elevation of CD86^+^ positive macrophages was reversed by circCDKAL1 silencing and OVA-induced elevation of CD206^+^ positive macrophages was further elevated by circCDKAL1 silencing (Fig. 3K). In total, circCDKAL1 silencing promoted macrophage M2 polarization and suppressed macrophage M1 polarization in OVA-induced mice and in MH-S cells, which were co-cultured with OVA-induced HNEpCs.

### YTHDC1 facilitated the cytoplasmic output of m6A-modified circCDKAL1

**Fig. 4:**
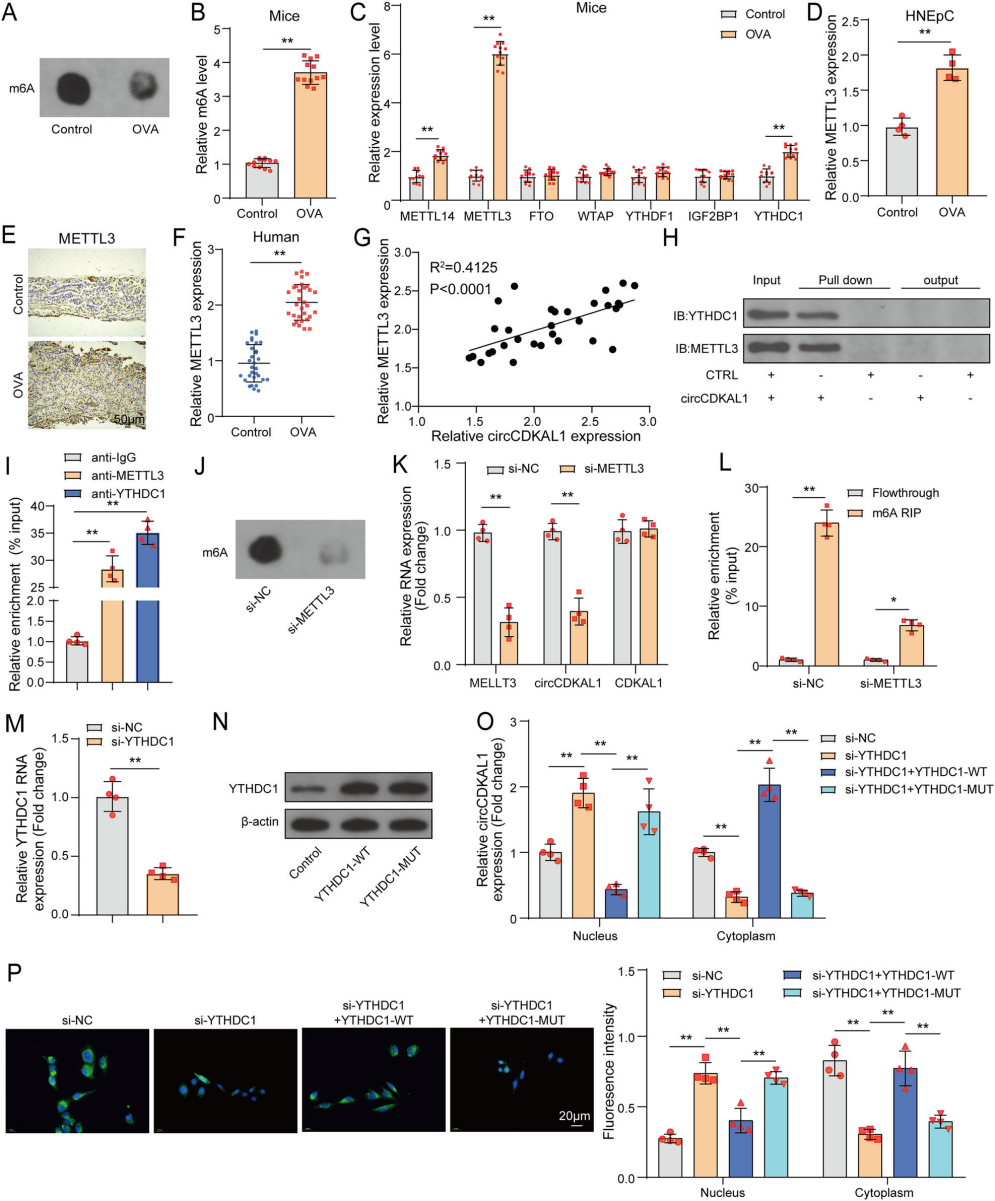
YTHDC1 facilitated the cytoplasmic output of m6A-modified circCDKAL1. **A**, **B** Total m6A level in the nasal mucosa of mice was detected using m6A dot blot assay and a commercial kit. *n* = 12. **C** The levels of related molecules of m6A modification in the nasal mucosa of mice were examined using RT-qPCR. *n* = 12. **D** METTL3 expression in OVA-induced HNEpCs was detected using RT-qPCR. *n* = 4. **E** METTL3 expression in nasal mucosa of AR patients was examined using IHC. *n* = 12. **F** METTL3 expression in NALF of AR patients was detected using RT-qPCR. *n* = 12. **G** The relationship between circCDKAL1 and METTL3 expression in NALF of AR patients was analyzed using Pearson correlation analysis. **H**, **I** The interaction between circCDKAL1 and METTL3/YTHDC1 in HNEpCs was validated by RNA pull-down and RIP. *n* = 4. **J** Total m6A level in HNEpCs transfected with si-METTL3 was detected using m6A dot blot assay. **K** CircCDKAL1 and linear CDKAL1 expression in HNEpCs with sh-METTL3 transfection was measured by RT-qPCR. *n* = 4. **L** m6A modification of circCDKAL1 in HNEpCs with sh-METTL3 transfection was determined using MeRIP. *n* = 4. **M** YTHDC1 expression in HNEpCs with si-YTHDC1 transfection was detected using RT-qPCR. *n* = 4. **N** YTHDC1 expression in HNEpCs with YTHDC1-WT or YTHDC1-MUT (MUT of m6A binding sites) transfection was evaluated using western blot. *n* = 4. **O**, **P** Distribution of circCDKAL1 in HNEpCs with si-YTHDC1 or together with YTHDC1-WT/YTHDC1-MUT transfection was detected using nuclear/cytoplasmic fractionation experiment and FISH assay. *n* = 4. *P < 0.05, **P < 0.01.

m6A modification is an important post-transcriptional regulation of genes [17]. Firstly, we found the higher level of total m6A in nasal mucosal tissues of OVA-induced mice (Fig. 4A, B). Then, the levels of m6A modification-related molecules including “writers”: METTL14, METTL3 and WTAP, “readers”: YTHDC1, IGF2BP1 and YTHDF1 and “erasers”: FTO were detected in nasal mucosal tissues of OVA-induced mice. The results exhibited that METTL14, METTL3 and YTHDC1 levels were elevated but the levels of other genes had no influences in mice with/without OVA induction (Fig. 4C). Meanwhile, METTL3 level was also apparently elevated in OVA-induced HNEpCs, nasal mucosal tissues and NALF of AR patients (Fig. 4D–F). Furthermore, there was a positive relationship between METTL3 and circCDKAL1 expression in NALF of AR patients (Fig. 4G). By conducting RNA pull-down, we found circCDKAL1 could interact with METTL3 and YTHDC1 in HNEpCs (Fig. 4H). METTL3 and YTHDC1 antibodies enriched observably circCDKAL1 in HNEpCs, which was determined by RIP (Fig. 4I). As expected, METTL3 silencing decreased total m6A level in HNEpCs (Fig. 4J). METTL3 silencing resulted in decreased circCDKAL1 expression but not linear CDKAL1 expression (Fig. 4K). Furthermore, METTL3 silencing reduced the level of total m6A level and m6A modified-circCDKAL1 (Fig. 4L). These above-mentioned results suggested that METTL3 silencing down-regulated circCDKAL1 expression through reducing m6A modified-circCDKAL1.

It was reported that YTHDC1 could promote cytoplasmic localization of m6A modified-circRNAs [18]. From Fig. 4H, I, there was an interaction between circCDKAL1 and YTHDC1 in HNEpCs. Here, si-YTHDC1 transfected into HNEpCs led to silenced YTHDC1 expression (Fig. 4M). In addition, constructing YTHDC1-WT and YTHDC1-MUT (MUT of m6A binding sites), western blot displayed that YTHDC1 expression was upregulated in HNEpCs with YTHDC1-WT and YTHDC1-MUT transfection (Fig. 4N). Nuclear/cytoplasmic fractionation experiment and FISH assay revealed that YTHDC1 knockdown decreased the level of circCDKAL1 in cytoplasm of HNEpCs, but increased in nucleus, while YTHDC1-WT reversed these alterations. Of note, YTHDC1-MUT had no influences on YTHDC1 knockdown-mediated distribution of circCDKAL1 in HNEpCs (Fig. 4O, P). Collectively, YTHDC1 promoted cytoplasmic localization of m6A mediated-circCDKAL1.

### METTL3 overexpression disrupted nasal epithelial cell adhesion and induced macrophage M1 polarization through enhancing circCDKAL1 expression

**Fig. 5:**
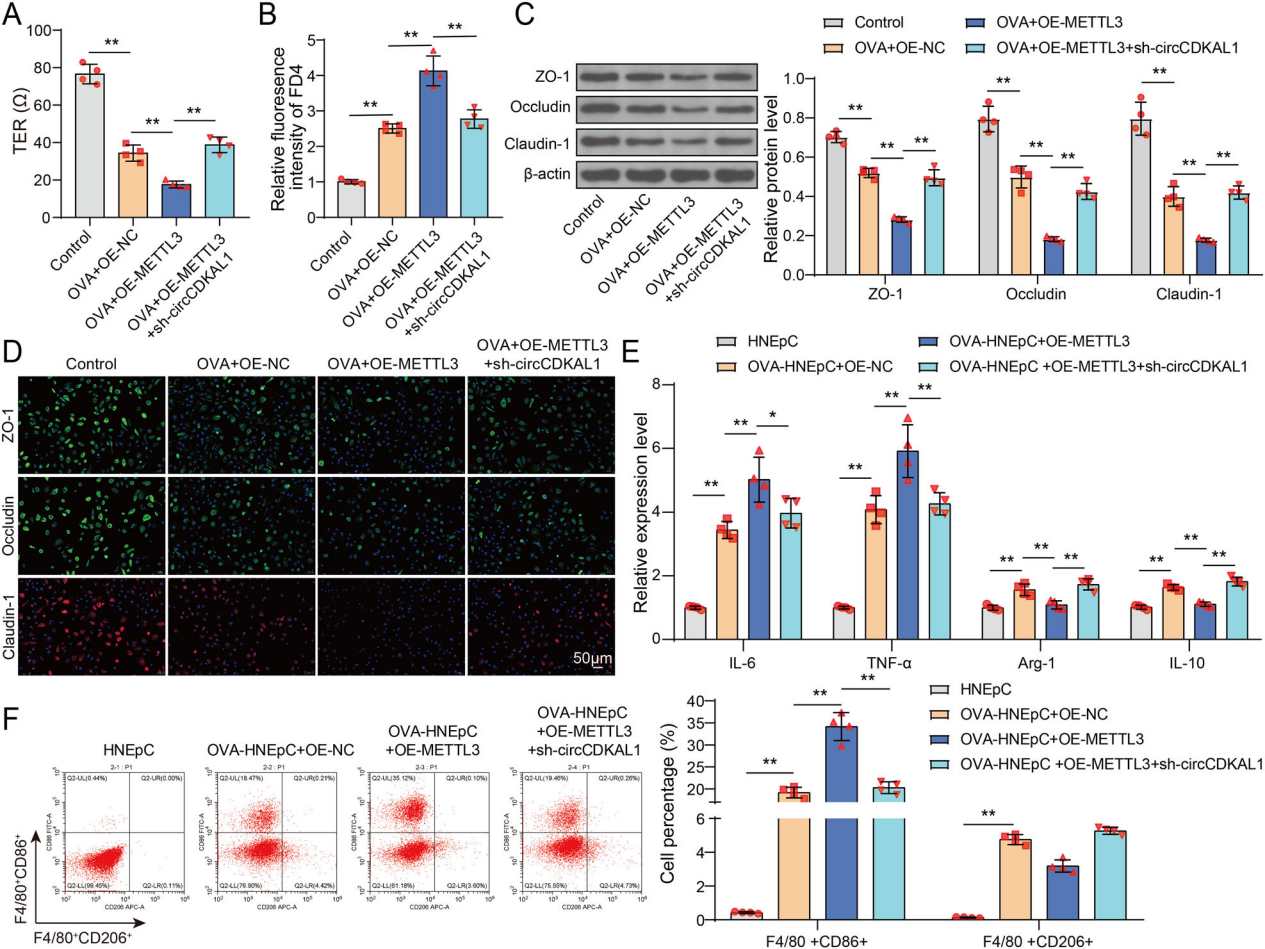
METTL3 overexpression disrupted nasal epithelial cell adhesion and induced macrophage M1 polarization through enhancing circCDKAL1 expression. HNEpCs were transfected with OE-METTL3 or together with sh-circCDKAL1 and followed by OVA treatment. *n* = 4. **A** TER was examined using EVOM/EndOhm system. *n* = 4. **B** FD4 levels in mice were evaluated using CLARIOstar. *n* = 4. **C**, **D** ZO-1, Occludin and Claudin-1 expression was detected using western blot and IF assay. *n* = 4. HNEpCs were transfected with OE-METTL3 or together with sh-circCDKAL1 and followed by OVA treatment, which was co-cultured with MH-S cells for 24 h. **E** The expression of IL-6, TNF-α, Arg-1and IL-10 in MH-S cells were examined by RT-qPCR. *n* = 4. **F** CD86^+^ and CD206^+^ positive macrophages were detected using flow cytometry. *n* = 4. *P < 0.05, **P < 0.01.

To elucidate the functions of METTL3/circCDKAL1 axis on maintaining nasal epithelial cell adhesion, HNEpCs were transfected with OE-METTL3 or together with sh-circCDKAL1 and followed by OVA treatment. METTL3 overexpression further strengthened OVA-induced reducing effect on TER in HNEpCs, whereas circCDKAL1 knockdown abolished this effect (Fig. 5A). OVA-induced FD4 level in HNEpCs was increased by METTL3 overexpression, however, circCDKAL1 knockdown reversed this elevation of FD4 level caused by METTL3 overexpression (Fig. 5B). OVA-induced reduction of ZO-1, Occludin and Claudin-1 expression in HNEpCs was further enhanced by METTL3 overexpression, whereas these phenomena were compromised by circCDKAL1 knockdown, which was evidenced by western blot and IF assay (Fig. 5C, D). Collectively, circCDKAL1 knockdown reversed METTL3 overexpression-mediated destruction of nasal epithelial cell adhesion of HNEpCs upon OVA induction.

As for influences of METTL3/circCDKAL1 axis on macrophage polarization, HNEpCs were transfected with OE-METTL3 or together with sh-circCDKAL1 and followed by OVA treatment, which were used to co-culture with MH-S cells for 24 h. OVA-induced elevation of IL-6 and TNF-α levels in MH-S cells was further reinforced by METTL3 overexpression and OVA-induced upregulation of Arg-1 and IL-10 levels in MH-S cells was abolished by METTL3 overexpression, but circCDKAL1 silencing neutralized METTL3 overexpression-mediated influences (Fig. 5E). Moreover, OVA-induced elevation of CD86^+^ positive macrophages was strengthened by METTL3 overexpression and OVA-induced elevation of CD206^+^ positive macrophages was offset by METTL3 overexpression, which was impaired by circCDKAL1 silencing (Fig. 5F). Taken together, circCDKAL1 knockdown abolished METTL3 overexpression-mediated promoting macrophage M1 polarization and suppressing macrophage M2 polarization in MH-S cells.

### CircCDKAL1 reduced the stability of JARID2 mRNA by interacting with IGF2BP2

**Fig. 6:**
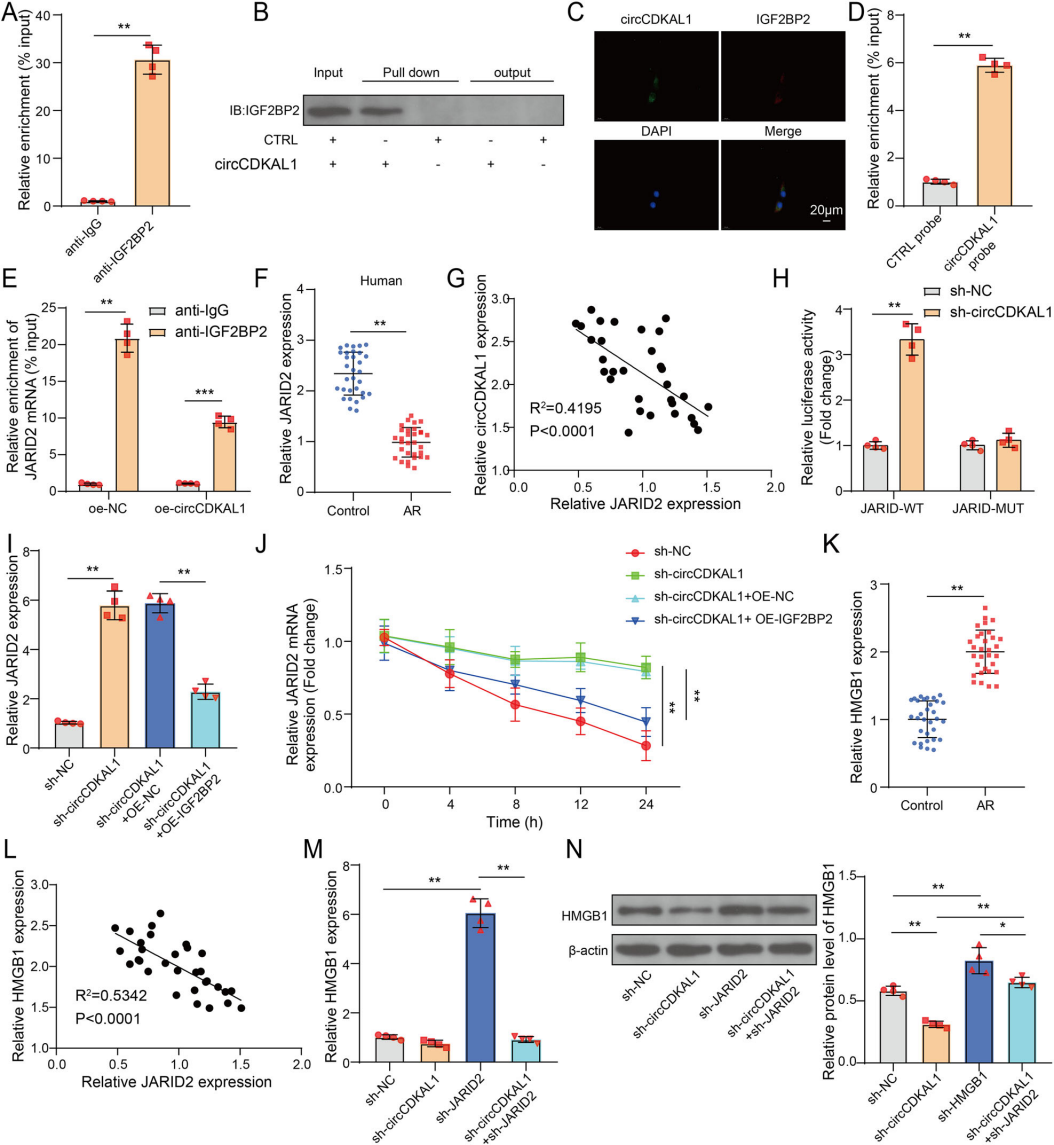
CircCDKAL1 reduced the stability of JARID2 mRNA by interacting with IGF2BP2. **A**, **B** The interaction between circCDKAL1 and IGF2BP2 was validated using RIP and RNA pull-down. *n* = 4. **C** The co-localization of circCDKAL1 and IGF2BP2 in HNEpCs was determined using IF-FISH assay. *n* = 4. **D** The interaction between JARID2 and circCDKAL1 was verified by RNA pull-down. *n* = 4. **E** The interaction between JARID2 and IGF2BP2 was verified by RIP. *n* = 4. **F** JARID2 expression in NALF of AR patients was detected using RT-qPCR. *n* = 4. **G** The relationship between JARID2 and circCDKAL1 in NALF of AR patients was analyzed using Pearson correlation analysis. **H** The interaction between JARID2 and circCDKAL1 was validated using a luciferase activity experiment. *n* = 4. **I**, **J** JARID2 expression and mRNA stability in HNEpCs with sh-circCDKAL1 transfection or together with OE-IGF2BP2 transfection were detected using RT-qPCR. *n* = 4. **K** HMGB1 expression in NALF of AR patients was evaluated using RT-qPCR. *n* = 4. **L** The relationship between HMGB1 and JARID2 expression in NALF of AR patients was analyzed using Pearson correlation coefficient. HNEpCs were transfected with sh-circCDKAL1 or/and sh-JARID2. **M**, **N** HMGB1 expression was measured using RT-qPCR and western blot. *n* = 4. *P < 0.05, **P < 0.01 and ***P < 0. 001.

Subsequently, the downstream molecules of circCDKAL1 were investigated in AR. It was reported that circRNAs interacted with RBPs, such as IGF2BP2, to affect mRNA stability of genes [19]. Here, Starbase website predicted that there harbored the binding site between circCDKAL1 and IGF2BP2. Furthermore, IGF2BP2 antibody observably enriched circCDKAL1 expression and circCDKAL1 probe also pulled down IGF2BP2 protein, which were evidenced by RIP and RNA pull-down, respectively (Fig. 6A, B). IF-FISH assay revealed that there was colocalization of circCDKAL1 and IGF2BP2 in cytoplasm mainly (Fig. 6C). JARID2 was reported to be related to airway inflammation [15]. Starbase website also predicted that there were binding site between JARID2 and IGF2BP2. Here, RNA pull-down exhibited the enrichment of JARID2 by circCDKAL1 probe (Fig. 6D). Additionally, RIP assay displayed the enrichment of JARID2 by IGF2BP2 antibody (Fig. 6E). Our results showed downregulation of JARID2 expression in NALF of AR patients (Fig. 6F), which was negatively related to circCDKAL1 expression (Fig. 6G). Luciferase activity experiment showed that circCDKAL1 knockdown promoted the luciferase activity in JARID2-WT group, but did not affect the luciferase activity in JARID2-MUT group (Fig. 6H). From Fig. 6D, E and Fig. 6G, the interactions among circCDKAL1, JARID2 and IGF2BP2. To investigate the influences of circCDKAL1/IGF2BP2 axis on JARID2 expression and JARID2 mRNA stability, HNEpCs were subjected to sh-circCDKAL1 transfection or together with OE-IGF2BP2 transfection. CircCDKAL1 silencing led to increased JARID2 expression, which was attenuated by IGF2BP2 overexpression (Fig. 6I). CircCDKAL1 silencing and IGF2BP2 silencing enhanced JARID2 mRNA stability, whereas IGF2BP2 overexpression eliminated circCDKAL1 silencing-mediated promotion of JARID2 mRNA stability (Fig. 6J and Supplementary Figure 1B). HMGB1 was widely reported in AR progression [20]. Figure 6K showed an abnormally high expression of HMGB1 in NALF of AR patients, which was negatively correlated with JARID2 expression (Fig. 6L). To clarify whether HMGB1 expression is affected by circCDKAL1/JARID2 axis, HNEpCs were transfected with sh-circCDKAL1 or/and sh-JARID2. HMGB1 expression was observably elevated by JARID2 silencing but evidently decreased by circCDKAL1 silencing and circCDKAL1 silencing reduced JARID2 silencing-mediated elevation of HMGB1 expression in HNEpCs (Fig. 6M, N). CircCDKAL1 interacted with IGF2BP2 to weaken JARID2 mRNA stability and inhibit JARID2 expression, which negatively regulated HMGB1 expression.

### CircCDKAL1 disrupted nasal epithelial cell adhesion and induced macrophage M1 polarization through regulating JARID2-mediated upregulation of HMGB1 expression

**Fig. 7:**
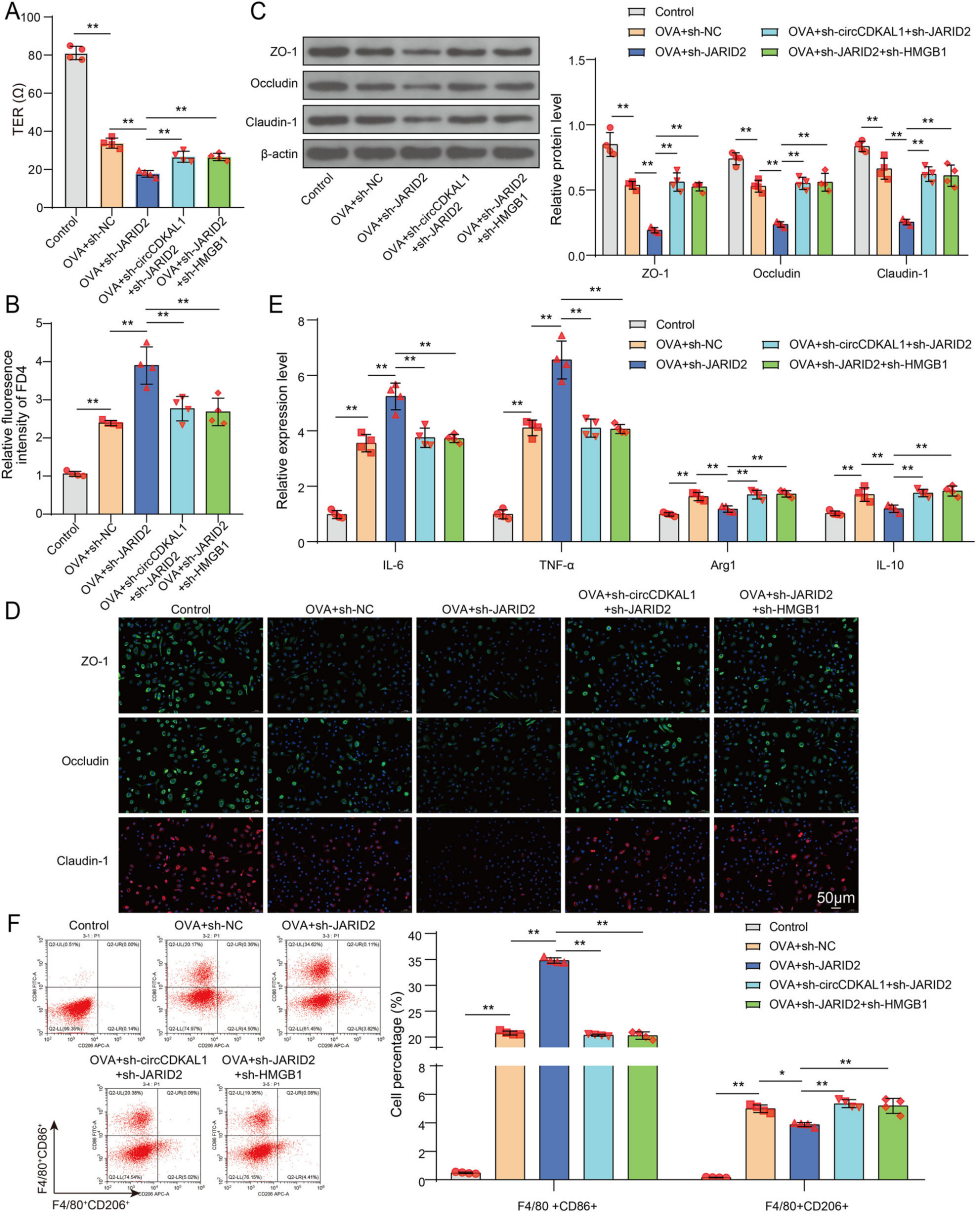
CircCDKAL1 disrupted nasal epithelial cell adhesion and induced macrophage M1 polarization through regulating JARID2-mediated upregulation of HMGB1 expression. HNEpCs were transfected with sh-JARID2 or together with sh-circCDKAL1 or sh-HMGB1 and followed by OVA treatment. **A** TER was examined using EVOM/EndOhm system. *n* = 4. **B** FD4 levels in mice were evaluated using CLARIOstar. *n* = 4. **C**, **D** ZO-1, Occludin and Claudin-1 expression was detected using western blot and IF assay. *n* = 4. HNEpCs were transfected with sh-JARID2 or together with sh-circCDKAL1 or sh-HMGB1 and followed by OVA treatment, which were co-cultured with MH-S cells for 24 h. **E** The expression of IL-6, TNF-α, Arg-1and IL-10 in MH-S cells were examined by RT-qPCR. *n* = 4. **F** CD86^+^ and CD206^+^ positive macrophages were detected using flow cytometry. *n* = 4. *P < 0.05, **P < 0.01.

To understand the functions of circCDKAL1/JARID2/HMGB1 axis on maintaining nasal epithelial cell adhesion, HNEpCs were transfected with sh-JARID2 or together with sh-circCDKAL1 or sh-HMGB1 and followed by OVA treatment. JARID2 knockdown further lowered TER in OVA-induced HNEpCs. However, combination of JARID2 knockdown and circCDKAL1/HMGB1 knockdown increased TER level compared to JARID2 knockdown solely (Fig. 7A). In OVA-induced HNEpCs, JARID2 knockdown enhanced FD4 level, while combination of JARID2 knockdown and circCDKAL1/HMGB1 knockdown resulted in lower FD4 level in relative to JARID2 knockdown alone (Fig. 7B). Western blot and IF assay exhibited that JARID2 silencing suppressed ZO-1, Occludin and Claudin-1 expression in OVA-induced HNEpCs. As expected, combination of JARID2 knockdown and circCDKAL1/HMGB1 knockdown contributed to promote the expression of these molecules compared to JARID2 knockdown alone (Fig. 7C, D). In total, circCDKAL1 knockdown enhanced nasal epithelial cell adhesion of OVA-induced HNEpCs through regulating JARID2/HMGB1 axis.

In terms of impacts of circCDKAL1/JARID2/HMGB1 axis on macrophage polarization, HNEpCs were transfected with sh-JARID2 or together with sh-circCDKAL1 or sh-HMGB1 and followed by OVA treatment, which were co-cultured with MH-S cells for 24 h. JARID2 knockdown elevated IL-6 and TNF-α levels in MH-S cells and reduced Arg-1 and IL-10 levels, but combination of JARID2 knockdown and circCDKAL1/HMGB1 silencing attenuated JARID2 knockdown-mediated these influences (Fig. 7E). Besides, JARID2 knockdown resulted in an elevation of CD86^+^ positive macrophages and a reduction of CD206^+^ positive macrophages. However, compared to the influences of JARID2 knockdown alone, combination of JARID2 knockdown and circCDKAL1/HMGB1 silencing resulted in lower percentage of CD86^+^ positive macrophages and higher percentage of CD206^+^ positive macrophages (Fig. 7F). Taken together, circCDKAL1 knockdown attenuated macrophage M1 polarization and promoted macrophage M2 polarization in MH-S cells.

## Discussion

AR is a common chronic disease worldwide, which is often associated with asthma, chronic sinusitis, conjunctivitis, etc. [1]. Deep understanding the pathogenesis of AR contributes to seeking more effective therapy. In this study, we found that circCDKAL1, with m6A modification mediated by METTL3, was abnormally high expression in AR patients and OVA-induced mice and HNEpCs. In addition, YTHDC1 promoted m6A modified-circCDKAL1 from nucleus to cytoplasm of HNEpCs. CircCDKAL1 in cytoplasm interacted with RBP IGF2BP2 to destabilize JARID2 mRNA and decrease JARID2 expression, which negatively regulated HMGB1, eventually weakening nasal epithelial cell barrier function, promoting macrophage M1 polarization and attenuating macrophage M2 polarization in AR.

The epithelial barrier formed by nasal epithelial cells is the first line of defense that protects the host immune system from detrimental stimuli. Its dysfunction, mainly manifesting in the damage of epithelial tight junction and the increase of epithelial permeability, was thought to be the pathogenesis of AR [21–23]. Massive studies have focused on the influences of various molecular regulatory mechanisms on the barrier function of nasal epithelial cells in AR [24, 25]. For instance, alpha-linolenic acid could restore the function of nasal mucosa epithelial barrier in AR by arresting CD4 + T cell differentiation via regulating IL-4Rα-JAK2-STAT3 pathway [24]. In addition, macrophages are an important part of the immune system, they can act as antigen-presenting cells and also produce cytokines and inflammatory mediators to help regulate the allergic immune response [26, 27]. Growing research has reported that macrophages are heterogeneous, and M1 or M2 polarization of macrophages plays crucial roles in various diseases, including AR [16, 28]. For instance, lncRNA GAS5, derived from exosomes, promoted macrophage M1 polarization via regulating mTORC1/ULK1/ATG13/NF-кB axis, thus aggravating AR [29]. Therefore, our results investigated the effects and molecular mechanism of circCDKAL1 on epithelial cell barrier function and macrophage polarization in AR.

CircRNAs are abundant in the eukaryotic transcription group [30]. Growing evidence has demonstrated that abnormal expression of circRNAs was implicated in progression of numerous diseases including AR [7, 31]. For example, circ_0067835 promoted an allergic inflammatory response in AR via regulating miR-155/GATA3 axis [6]. CircCDKAL1, was found to be abnormally higher expression in AR patients via HTS [8]. However, the functions and underlying regulatory mechanism of circCDKAL1 in diseases including AR had no reports. In this study, firstly, we validated that circCDKAL1 possessed a loop structure and its location located in cytoplasm mainly. Secondly, we determined abnormally higher expression of circCDKAL1 in samples of AR patients and OVA-induced mice and HNEpCs. Thirdly, we found that, OVA resulted damage of nasal mucosal epithelial barrier function/epithelial cell adhesion, promotion of macrophage M1 polarization and suppression of macrophage M2 polarization in mice and HNEpCs, however, circCDKAL1 knockdown partially reversed these phenomena.

m6A modification is a common post-transcriptional modification of mRNA and ncRNA including circRNA [32], which mediates post-transcriptional mRNA metabolisms such as mRNA splicing, mRNA stability, mRNA translation efficiency, and mRNA nuclear export [33–36]. In general, circRNAs are co-transcripts of the typical linear mRNA splicing that occurs in the nucleus [11]. However, most circRNAs were mainly located in the cytoplasm. Therefore, elucidating the potential mechanism of circRNAs on exporting from the nucleus to the cytoplasm is a key issue to understand the role of circRNA in physiological and pathological situations. Encouragingly, there was a mechanism reporting that circRNAs could be modified by m6A and YTHDC1, m6A “reader” protein, could shift m6A-modified mRNA or circRNAs from nucleus to the cytoplasm [33, 37]. For example. Dawei Rong et al. pointed out that YTHDC1 facilitated cytoplasmic output of m6A-modified circHPS5, which was mediated by METTL3 [38]. In this study, we found that METTL3 mediated m6A modification of circCDKAL1 to positively affect circCDKAL1 expression through interacting with circCDKAL1. Additionally, YTHDC1 promoted the output of circCDKAL1 in cytoplasm through interacting with circCDKAL1, which process was dependent on circCDKAL1 being in m6A modification. Furthermore, circCDKAL1 silencing abolished METTL3 overexpression-mediated destruction of epithelial cell adhesion, promotion of macrophage M1 polarization and suppression of macrophage M2 polarization in OVA-induced HNEpCs.

According to previous researches, circRNAs participated in the occurrence and development of diseases through different molecular regulatory mechanisms downstream [39]. Among them, circRNA interacted with RBP to affect the stability of downstream genes, which has been widely studied [13]. IGF2BP2, an RBP, was widely reported to interact with circRNAs, thus mediating stabilization of targeted genes’ mRNA [19, 40]. For instance, circ-TNPO3 bound to IGF2BP2, resulting in destabilization of SERPINH1 mRNA in clear cell renal cell carcinoma [19]. Here, we validated the interactions among circCDKAL1, IGF2BP2 and JARID2, eventually attenuating the stability of JARID2 mRNA and suppressed JARID2 expression in HNEpCs. JARID2 was reported to be related to airway inflammation [15]. However, the role of JARID2 in AR had no reports. In our study, we found that JARID2 expression was evidently decreased in samples of AR patients, which was negatively related to circCDKAL1 expression. HMGB1 was regarded as an inflammatory mediator initiator, which was reported to be upregulated in AR patients [41]. Similarly, in current study, HMGB1 expression was observably elevated in samples of AR patients and was negatively related to JARID2 expression. Furthermore, we found that circCDKAL1 silencing resulted in decreased JARID2 expression, but JARID2 silencing reversed this alteration. Moreover, JARID2 silencing contributed to destruction of epithelial cell adhesion, promotion of macrophage M1 polarization and suppression of macrophage M2 polarization in OVA-induced HNEpCs, however, combination of JARID2 knockdown and circCDKAL1/HMGB1 silencing weakened the influences of JARID2 silencing alone.

However, our study also has some limitations. First, we only discussed the upstream regulation of circCDKAL1 from the aspect of circCDKAL1m6A modification, and did not pay attention to more regulatory modes. Second, there are many types of ways involved in the regulation of downstream gene expression by circRNA, and we only discussed the interaction of circCDKAL1 and RBP. Third, we did not deeply explore how circCDKAL1 and IGF2BP2 affect JARID2 mRNA? Fourth, how JARID2 negatively regulates HMGB1 expression and then participates in the pathological process of AR has not been discussed. In the future, we will further explore the molecular regulatory mechanism of circCDKAL1 in AR.

## Conclusion

**Fig. 8:**
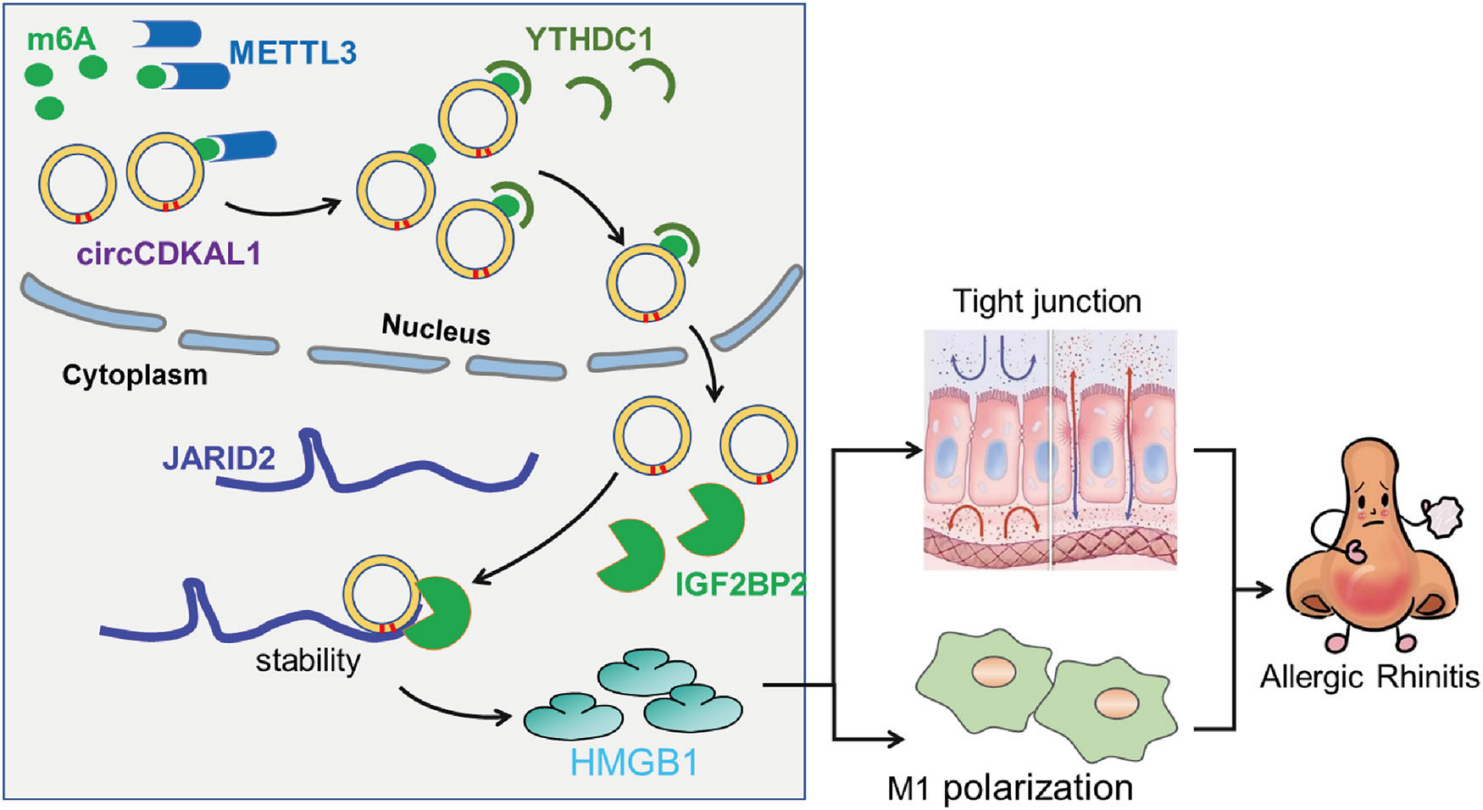
Schematic diagram of mechanism. circCDKAL1, which was regulated by METTL-mediated m6A modification and was transferred from the nucleus to the cytoplasm by YTHDC1 depending on m6A modification, impaired nasal mucosal epithelial barrier function/epithelial cell adhesion and promoted M1 macrophage polarization in AR through interacting with IGF2BP2 and regulating JARID2/HMGB1 axis.

In summary, the above-mentioned evidence illustrated that METTL3 mediated m6A modification of circCDKAL1 to elevate circCDKAL1 expression and YTHDC1 transferred circCDKAL1 from the nucleus to the cytoplasm in a m6A modified manner in AR. Additionally, circCDKAL1 impaired nasal mucosal epithelial barrier function/epithelial cell adhesion, promoted macrophage M1 polarization and inhibited macrophage M2 polarization in AR through interacting with IGF2BP2 and regulating JARID2/HMGB1 axis (Fig. 8).

## Methods

### Collection of the clinical specimens

A total of 31 cases of AR patients and 31 cases of healthy volunteers were recruited in the Hainan Affiliated Hospital of Hainan Medical University. Inclusion criteria were as follows: firstly, all participants received no other treatments and all healthy participants had no airway symptom and had negative sIgE results; Secondly, the included AR patients exhibited a positive skin-prick test depended on guidelines for AR [42]; Thirdly, the AR patients were enrolled in more than 1-year AR course and the ages of AR patients were within the range of 18–60 years old in this study; Fourthly, all AR patients were not subjected to corticosteroid therapy 1 month before recruitment. In addition, exclusion criteria of all participants were as follows: (1) with chronic sinusitis, nasal polyps, asthma, atopic dermatitis, immunodeficiency or other diseases related to immune abnormality; (2) taking glucocorticoids and/or antihistamines within 2 weeks; (3) pregnancy; (4) uncooperative participants. All recruited participants underwent nasal lavage and then their nasal lavage fluid (NALF) samples were collected for subsequent experiments. In addition, nasal mucosal tissues were collected from recruited participants for subsequent experiments. All samples were stored at −80 °C. The experimental procedures were approved by the Ethics Committee of the Hainan Affiliated Hospital of Hainan Medical University (Approval No.: Med-Eth-Re [2024]246). Informed consents were signed by all participants.

### AR mice model and treatment

Male C57BL/6 mice (aged 6 weeks) were acquired from Slac Jingda Laboratory Animal Co., Ltd. (Hunan, China). After one week of adaptive feeding, all mice were officially used as experimental subjects to start the intervention. Firstly, mice were randomly divided into two groups: Control and ovalbumin (OVA). Each group contained 12 mice. OVA group referred to the group that simulated AR. Of note, OVA group mice were treated with the following procedures: mice were intraperitoneally injected by 25 μg OVA and 1 mg aluminum hydroxide gel at 0, 7, and 14 d, respectively, so that the mice were in the basal sensitization stage. Then, basal sensitized mice were challenged with 500 μg OVA from day 21 to day 27 via intranasal exposure to mimic AR pathological process. To explore the role of circCDKAL1 in AR mice, mice were randomly divided into three groups: Control, OVA+sh-NC, OVA+sh-circCDKAL1. Each group contained 12 mice. In OVA+sh-NC or OVA+sh-circCDKAL1 group. 1 × 10^7^ TU/mL lentiviruses carrying sh-NC or sh-circCDKAL1 (GenePharma, China) were injected into basal sensitized mice through a tail vein on days 19 and 20 d. After the OVA intranasal challenge, frequencies of sneezing and nasal rubbing within 15 min were documented. At 28 d, mice were sacrificed. Tissue samples and NALF from mice were collected for follow-up experiments. All animal procedures were in accordance with National Institutes of Health guidelines and approved by the Animal Care and Use Committee of Hainan Affiliated Hospital of Hainan Medical University (Approval No.: Med-Eth-Re [2024]246). Of note, Gpower was conducted to choose sample size. Random allocation was performed using the table of random number, and experiments involving mice were conducted in a blind manner. The investigator was blinded to the group allocation during experiments.

### Cell culture and establishment of an AR cell model

Human nasal epithelial cells (HNEpCs) were obtained from PromoCell GmbH (PromoCell, Heidelberg, Germany). Cells were authenticated by STR profiling and tested for mycoplasma contamination. HNEpCs were grown in airway epithelial cell growth medium (PromoCell). MH-S cells, belong to a mouse-derived alveolar macrophage line, were obtained from the National Infrastructure of Cell Line Resource (China). MH-S cells were cultured in DMEM (Gibco, USA). The above-mentioned media were supplemented with 10% fetal bovine serum (FBS, Gibco), 100 U/ml penicillin and 0.1 mg/ml streptomycin (Beyotime, China). All cells were cultured under the condition of 5% CO_2_ and 37 °C. 2 μg/ml OVA was applied for incubation of HNEpCs for 24 h to establish an AR cell model.

### Cell transfection

The small interfering RNA targeting METTL3 (si-METTL3), YTHDC1 (si-YTHDC1), the short hairpin RNA targeting JARID2 (sh-JARID2), HMGB1 (sh-HMGB1), circCDKAL1 (sh-circCDKAL1), IGF2BP2 (sh-IGF2BP2), overexpression vector of METTL3 (OE-METTL3), IGF2BP2 (OE-IGF2BP2) and their correspondingly negative controls including si-NC, sh-NC, OE-NC were obtained from GenePharma (Shanghai, China). For YTHDC1-wild type (YTHDC1-WT, sequence: GAACU) and YTHDC1 mutant (YTHDC1-MUT, sequence: CUUGA), YTHDC1-WT were synthesized and subcloned into the psiCHECK2 vector (GenePharma) and YTHDC1-MUT, a plasmid with a mutated sequence in its m6A site, was generated by site-directed mutagenesis (GenePharma). For cell transfection, HNEpCs were seeded onto 6-well plates and incubated overnight. Then, cells were transfected with above sequences or plasmids for 48 h using Lipofectamine™ 3000 (Invitrogen, USA) following the instructions.

### Sanger sequencing

The backsplicde site of circCDKAL1 was verified by divergent primers. Sanger sequencing was performed by Tsingke (China).

### Agarose gel electrophoresis

According to previously documented, designing divergent primers and convergent primers were used to amplify cDNA and gDNA. Subsequently, the PCR products were analyzed using agarose gel electrophoresis [43, 44].

### RNase R digestion

To detect the degradation of circCDKAL1 and linear CDKAL1, RNAs from HNEpCs were treated with RNase R (3 U/µg, Sigma-Aldrich, USA) for 1 h at 37 °C. Subsequently, RT-qPCR was used to detect the levels of circCDKAL1 and linear CDKAL1 expression.

### RNA stability

To detect RNA stability of circCDKAL1 and linear CDKAL1, 2 μg/mL actinomycin D was used to block gene transcription process of HNEpCs. RT-qPCR was applied to evaluate the levels of circCDKAL1 and linear CDKAL1 after HNEpCs were treated with 2 μg/mL actinomycin D for 0, 6, 12 and 24 h, respectfully. For JARID2 mRNA stability, after HNEpCs were treated with 2 μg/mL actinomycin D for 0, 4, 8, 12, 24 h, respectfully. RT-qPCR was used to detect the level of JARID2 mRNA in different time points.

### Nuclear/cytoplasmic fractionation

Nuclear and cytoplasmic RNAs of HNEpCs were separated using PARIS™ Kit (Invitrogen, USA). Then, the expression of circCDKAL1 in cytoplasm and nucleus was detected by RT-qPCR.

### RT-qPCR

Total RNA was extracted from clinical samples, mice, macrophagocytes and HNEpCs using TRIzol reagent (Beyotime). cDNA synthesis was performed using a Script Reverse Transcription Reagent Kit (TaKaRa, China). SYBR Premix Ex Taq II Kit (TaKaRa) was used for qPCR process. RT-qPCR was processed in the ABI 7500 Real-Time PCR System (Thermofisher, USA). The primer sequences were shown in Table 1. Targeted genes’ relative expression was computed using 2 − ΔΔCt formula. GAPDH served as a reference gene.

**Table 1:** Primer sequences in RT-qPCR.

Gene name	5’-3’
circCDKAL1 (human)	Forward: ACGAACATGGGGTTGTTCTCA
	Reverse: ACGAACATGGGGTTGTTCTCA
circCDKAL1 (mouse)	Forward: TGGATACGAACATGGGGTTGT
	Reverse: CCAGTAGTGTATCACAGGATGC
CDKAL1 (human)	Forward: TTCTTGACCGACTGAGACCCA
	Reverse: TCATGTTCTCCAACGCCTCTT
CDKAL1 (mouse)	Forward: CTCTCAGTTCGGACAGATTCATCTTTCAAGAGGAC
	Reverse: GCTGTGATCTGGTCTGAGGTCAGATGGC
IL-6 (human)	Forward: GTAGCCGCCCCACACAGA
	Reverse: CATGTCTCCTTTCTCAGGGCTG
IL-6 (mouse)	Forward: CAAAGCCAGAGTCCTTCAGAG
	Reverse: AGCATTGGAAATTGGGGTAG
TNF-α (human)	Forward: TGGCCCAGACCCTCACACTCAG
	Reverse: ACCCATCGGCTGGCACCACT
TNF-α(mouse)	Forward: TCTTCTCATTCCTGCTTGTGG
	Reverse: ATGAGAGGGAGGCCATTTG
Arg-1 (human)	Forward: CTGGCAAGGTGGCAGAAGTC
	Reverse: ATGGCCAGAGATGCTTCCAA
Arg-1 (mouse)	Forward: AAGAATGGAAGAGTCAGTGTGG
	Reverse: GGGAGTGTTGATGTCAGTGTG
IL-10 (human)	Forward: AAGGACCAGCTGGACAACAT
	Reverse: AGACACCTTTGTCTTGGAGCTTA
IL-10 (mouse)	Forward: TCACTCTTCACCTGCTCCAC
	Reverse: CTATGCTGCCTGCTCTTACTC
METTL14 (human)	Forward: GAACACAGAGCTTAAATCCCCA
	Reverse: TGTCAGCTAAACCTACATCCCTG
METTL14 (mouse)	Forward: TCACCTCCTCCCAAGTCCAAGTC
	Reverse: CCCTAAAGCCACCTCTCTCTCCTC
METTL3 (human)	Forward: AAGGAGCCGGCTAAGAAGTC
	Reverse: TCACTGGCTTTCATGCACTC
METTL3 (mouse)	Forward: CAGTGCTACAGGATGACGGCTT
	Reverse: CCGTCCTAATGATGCGCTGCAG
WTAP (human)	Forward: GGCGAAGTGTCGAATGCT
	Reverse: CCAACTGCTGGCGTGTCT
WTAP (mouse)	Forward: GGAAGTTTACGCCTGATAGCC
	Reverse: TCTGCTTCAAGCTGTGCAAT
YTHDC1 (human)	Forward: AACTGGTTTCTAAGCCACTGAGC
	Reverse: GGAGGCACTACTTGATAGACGA
YTHDC1 (mouse)	Forward: CCATCCCGTCGAGAACCAG
	Reverse: TGGTCTCTGGTGAAACTCAGG
IGF2BP1 (human)	Forward: CAGTCCAAGATAGACGTGCATAG
	Reverse: CTCAGGGTTGTAAAGGGTAAGG
IGF2BP1 (mouse)	Forward: TGAGACAGCGGTGGTCAACGTCAC
	Reverse: TCCTGGAGCGATGAGATGGTGATC
YTHDF1 (human)	Forward: CAAGCACACAACCTCCATCTTCG
	Reverse: GTAAGAAACTGGTTCGCCCTCAT
YTHDF1 (mouse)	Forward: GGACAGTCCAATCCGAGTAACA
	Reverse: CCTCGCTGAGGGAGTAAGGA
FTO (human)	Forward: GCCTCGGTTTAGTTCCACTCAC
	Reverse: GTCGCCATCGTCTGAGTCATTG
FTO (mouse)	Forward: TCACAGCCTCGGTTTAGTTC
	Reverse: GCAGGATCAAAGGATTTCAACG
JARID2 (human)	Forward: ACTTGTGCTACCTGTCCATG
	Reverse: TCGTAGCGGTACATCAGCTT
JARID2 (mouse)	Forward: GGTGCAGGTACAAACAGTGCCAAA
	Reverse: GTGGTGGTTGGGTTTGGTTTCCTT
HMGB1 (human)	Forward: TAACTAAACATGGGCAAAGGAG
	Reverse: TAGCAGACATGGTCTTCCAC
HMGB1 (mouse)	Forward: GGCGAGCATCCTGGCTTATC
	Reverse: GGCTGCTTGTCATCTGCTG
GAPDH (human)	Forward: CGGAGTCAACGGATTTGGTCGTAT
	Reverse: AGCCTTCTCCATGGTGGTGAAGAC
GAPDH (mouse)	Forward: CGACTTCAACAGCAACTCCCACTCTTCC
	Reverse: TGGGTGGTCCAGGGTTTCTTACTCCTT

### Fluorescence in situ hybridization (FISH)

To detect the location of circCDKAL1 in nasal mucosal tissue of mice and HNEpCs, FISH assay was conducted. Cy5-labeled circCDKAL1 probe was synthesized by Servicebio (China). Then, circCDKAL1 probe was applied to incubate samples. DAPI stained cell nuclei. Fluorescence intensity was examined using a fluorescence microscope (Olympus, Japan).

### Enzyme-linked immunosorbent assay (ELISA)

The levels of IgE, sIgE, IL-1β, IL-6, TNF-α, Arg-1and IL-10 in the NALF of AR patients or serum/NALF of mice were determined by ELISA kits following the manufacturer’s instructions. The kit of sIgE was purchased from Nanjing Jiancheng Bioengineering Insitute (China). The others were purchased from Abcam.

### Hematoxylin and eosin (HE) staining and periodic acid-Schiff (PAS) staining

After fixation with 4% paraformaldehyde and embeddedness with paraffin, nasal mucosal tissues of mice were cut into 5 µm thick serial sections. Partial sections were stained with hematoxylin and eosin (Beyotime). Others were stained with PAS (Beyotime) for analysis of blet cell hyperplasia. An optical microscope with a camera (Olympus, Japan) took pictures of the stained sections.

### Terminal deoxynucleotidyl transferase dUTP nick end labeling (TUNEL)

The nasal mucosal tissues from mice were fixed with 4% paraformaldehyde and permeabilized with 0.1% Triton X-100. After washing, TUNEL reaction mixture was incubated at 37 °C for 1 h, and the stained cells were observed with fluorescence microscopy (Eclipse TE300, Nikon, Japan).

### Fluorescein isothiocyanate-dextran (FD4) permeability assay

Nasal Epithelial permeability, which is related to nasal epithelial cell barrier function, was evaluated by FD4 permeability assay. FD4 was purchased from Sigma-Aldrich (USA). For FD4 level in mice, mice were administered FD4 through nostrils at 4 h before sacrifice and collected plasma of mice. For FD4 level in HNEpCs, 2 mg/mL FD4 was applied to the cells cultured in transwell inserts. FD4 level in the fluid on the basolateral side was detected after 4 h. FD4 level was detected using CLARIOstar (BMG LABTECH, Germany).

### Immunofluorescence (IF)

Referring to previous studies [45, 46], tissue samples and HNEpCs were prepared. After blocking with 5% BSA, the primary antibodies against ZO-1 (ab307799, Abcam), Occludin (ab216327, Abcam), Claudin-1(ab307692, Abcam), TPSAB1 (mast cell tryptase, PA5-102552, Thermo Fisher Scientific), CD68 (ab283654, Abcam), CD206 (MA5-16871, Thermo Fisher Scientific) and iNOS (PA1-036, Thermo Fisher Scientific) were applied to incubate samples overnight at 4 °C. Cy5-conjugated, Alexa 488-conjugated secondary antibodies (Thermo Fisher Scientific) were selected to incubate samples according to corresponding primary antibodies. Afterward, DAPI stained cell nucleus. The images were pictured using a fluorescence microscope.

### Measurement of transepithelial resistance (TER)

TER was used to evaluate nasal epithelial cell barrier function. In short, HNEpCs (1 × 10^5^ cells/per transwell) were first seeded on polyester transwell inserts (Corning, USA). After the cells reached complete confluence, TER was determined using an EVOM/EndOhm system (WPI Inc, USA).

### Western blot

RIPA lysis buffer (Beyotime, China) was adopted to acquire proteins from clinical samples, mice, macrophages and HNEpCs. SDS-PAGE was performed to segregate the proteins. Next, the separated proteins on SDS-PAGE were transferred onto PVDF membranes. After blocking by skimmed milk (5%), primary antibodies were applied to incubate the PVDF membranes overnight at 4 ˚C and HRP-conjugated secondary antibody (Beyotime) was conducted to incubate the membranes for 1 h. ECL kit (Beyotime) was used to visualize the protein bands. The densitometry analysis was evaluated by using ImageJ.

The primary antibodies used in this study were as follows: ZO-1(ab307799, 1:000, Abcam, British), Occludin (ab216327, 1:1000, Abcam), Claudin-1 (ab307692, 1:1000, Abcam), YTHDC1 (ab264375, 1:5000, Abcam), HMGB1 (ab18256, 1:1000, Abcam) and β-actin (ab8226, 1:1000, Abcam).

### Flow cytometry

After co-culture with HNEpCs for 24 h, MH-S cells were collected. M1 and M2 microphase markers were determined with flow cytometry (BD Biosciences, USA) employing FITC-labeled anti-CD86 (ab239075, Abcam), Alexa Fluor® 647-labeled anti-CD206 (MA5-16871, Thermo Fisher Scientific) and PE-labeled anti-F4/80 (MF48000, Thermo Fisher Scientific). The percentage of M1 or M2 microphases was measured using FlowJo 10 software (FlowJo, USA).

### m6A dot blot assay

In brief, the isolated total RNA was denatured at 65 °C for 5 min and then combined with saline sodium citrate (SSC) buffer. The RNA samples were subsequently transferred onto an Amersham Hybond-N+ membrane (Solarbio, China). After UV crosslinking for 5 min, the membranes were stained with 0.02% methylene blue (Coolaber, China) and scanned for imaging as a loading control. Alternatively, the UV-crosslinked membranes were blocked with 5% skim milk for over 1 h and incubated with anti-m6A antibody (MA5-33030, Thermo Fisher Scientific) overnight at 4 °C. The dot blot signals were detected using an imaging system following incubation with the secondary antibody.

### m6A RNA methylation quantification

To measure the levels of m6A in nasal mucosa of mice, an EpiQuik m6A RNA Methylation Quantitation Kit (Epigentek, USA) was used to analyzes of m6A levels through colorimetric method following product manual.

### Immunohistochemistry (IHC)

5 μm sections of nasal mucosal tissues of mice were prepared. After repairing the antigen, the sections were blocked with 1% BSA. The sections were incubated with antibody against METTL3 (Abcam) overnight at 4 °C and then incubated with HRP-labelled antibody. The sections were counterstained with diaminobenzidine (DAB). Finally, the images were acquired using an optical microscope (Japan).

### RNA immunoprecipitation (RIP) assay

HNEpCs were lysed with RIP Lysis Buffer. The supernatants were obtained after centrifugation. The magnetic beads conjugated with anti-METTL3 (ab195352, Abcam), anti-KLF9 (ab227920, Abcam), anti-YTHDC1 (ab264375, Abcam), IGF2BP2 (ab128175, Abcam) or Ig G (ab172730, Abcam) were added into the supernatants. IgG acted as the control. The beads-bound complexes of RNA were eluted with elution buffer. Then, immune-precipitated RNAs were determined using RT-qPCR.

### RNA pull-down

HNEpCs were transfected with a circCDKAL1 probe. 48 h later, cells were incubated with lysis buffer after washing. The lysate was incubated with streptavidin-coated magnetic beads at 4 °C overnight. The enrichment of YTHDC1, METTL3 and IGF2BP2 was examined by western blot.

### M6A immunoprecipitation (MeRIP)

Total RNA was isolated from HNEpCs using Trizol regent. Anti-m6A (ab208577, Abcam) or anti-IgG (ab172730, Abcam) conjugated with A/G magnetic beads was applied for incubating RNA samples. Subsequently, RNA was precipitated by beads and then was eluted. The precipitated RNA was detected by RT-qPCR.

### IF-FISH assay

According to a previous study, we conducted an IF-FISH assay [47]. Briefly, HNEpCs were fixed with 4% paraformaldehyde and permeabilized with 0.5% Triton X-100. Then, IGF2BP2 (ab124930, Abcam) and fluorescent-tagged secondary antibodies were used to incubate cells. After the antibody incubation, HNEpCs were fixed with 4% paraformaldehyde and dehydrated in an ethanol series of 70%, 95%, and 100%. The dehydrated cells were then hybridized with Cy5-labeled circCDKAL1 probe. Confocal images were acquired with a fluorescence microscope (Carl Zeiss, USA).

### Dual-luciferase reporter assay

The interaction between circCDKAL and JARID2 3’ UTR was validated using a dual luciferase reporter assay. JARID2 3’ UTR wild type (JARID2-WT: AGGAGTTC) and mutant (JARID2-MUT: GTTCACCA) were obtained by GenePharma. sh-circCDKAL/sh-NC in combination with JARID2-WT/JARID2-MUT using Lipofectamine 3000 (Invitrogen, USA) were transfected into HNEpCs. Dual luciferase reporter system kit (Promega, USA) was employed for evaluating luciferase activity.

### Statistical analysis

All data were presented as means ± standard deviation (SD). Using GraphPad Prism 9 (Graphpad, USA) analyzed all data. Student’s t-test was applied to analyze comparison of two groups and a one-way analysis of variance (ANOVA) followed by post hoc tests was applied to conduct a comparison of more than two groups. Non-parametric analysis was performed using Kruskal-Wallis test with Dunn’ multiple comparisons test. The relationships of genes were analyzed using Pearson correlation analysis. *p* < 0.05 was regarded as a statistically significant difference. Each experiment was performed at least 3 times.

## Supplementary information

Supplementary Material

Supplementary Figure 1

Supplementary Figure Legend

Supplementary File 1

Supplementary File 2

## Data Availability

The datasets used or analyzed during this study can be made available from the corresponding author upon reasonable request.
