# Supplementary figures and images for: METTL3-mediated m6A modification of circCDKAL1 regulates macrophage M1 polarization and nasal epithelial cell barrier function in allergic rhinitis through IGF2BP2/JARID2/HMGB1 axis
Source: Cell Death Discov. 2025 Aug 29;11:417. doi: 10.1038/s41420-025-02710-7 (PMC12397420; doi:10.1038/s41420-025-02710-7)

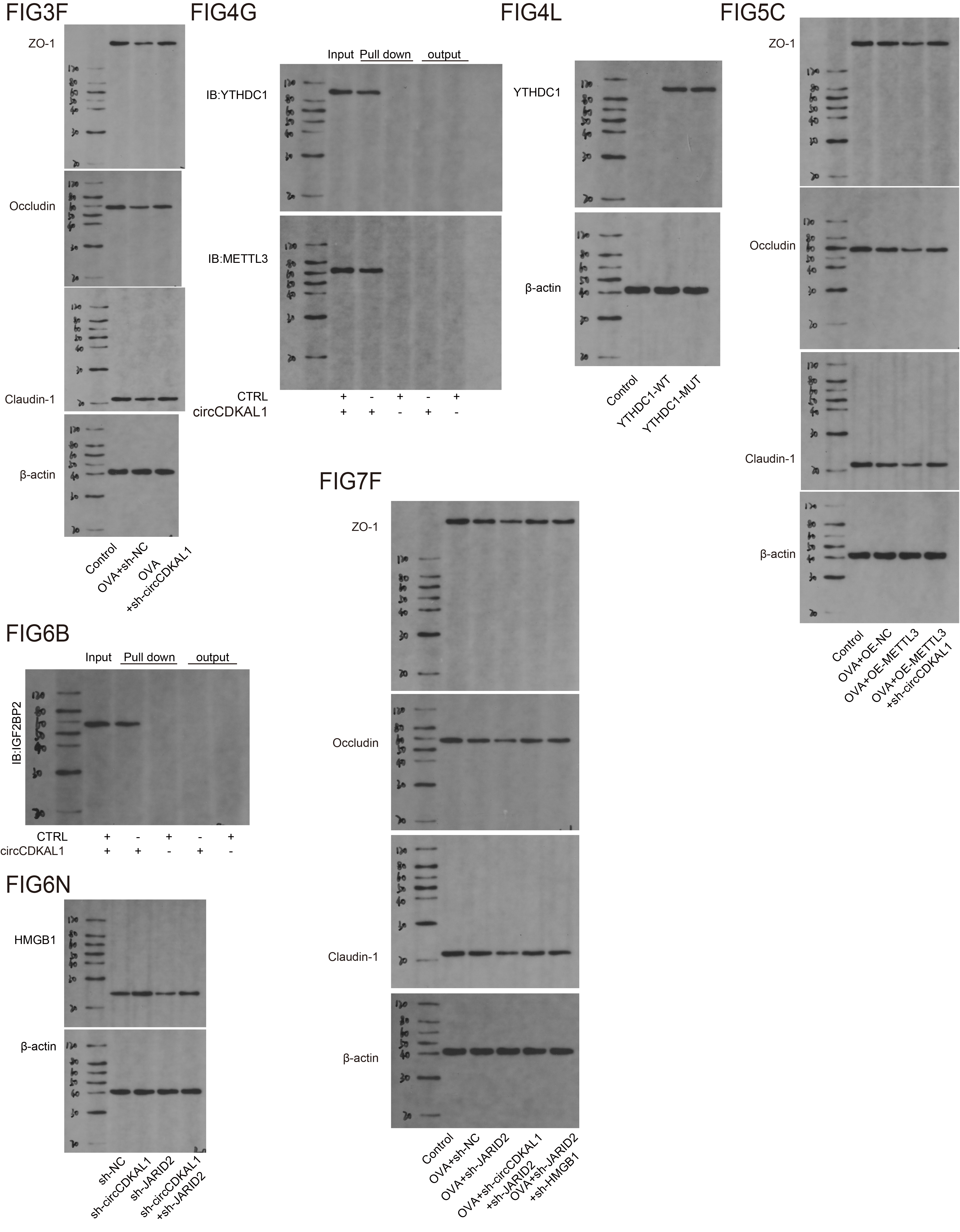

Supplement: Supplementary file 1 — Supplementary Material [file 41420_2025_2710_MOESM1_ESM.tif]

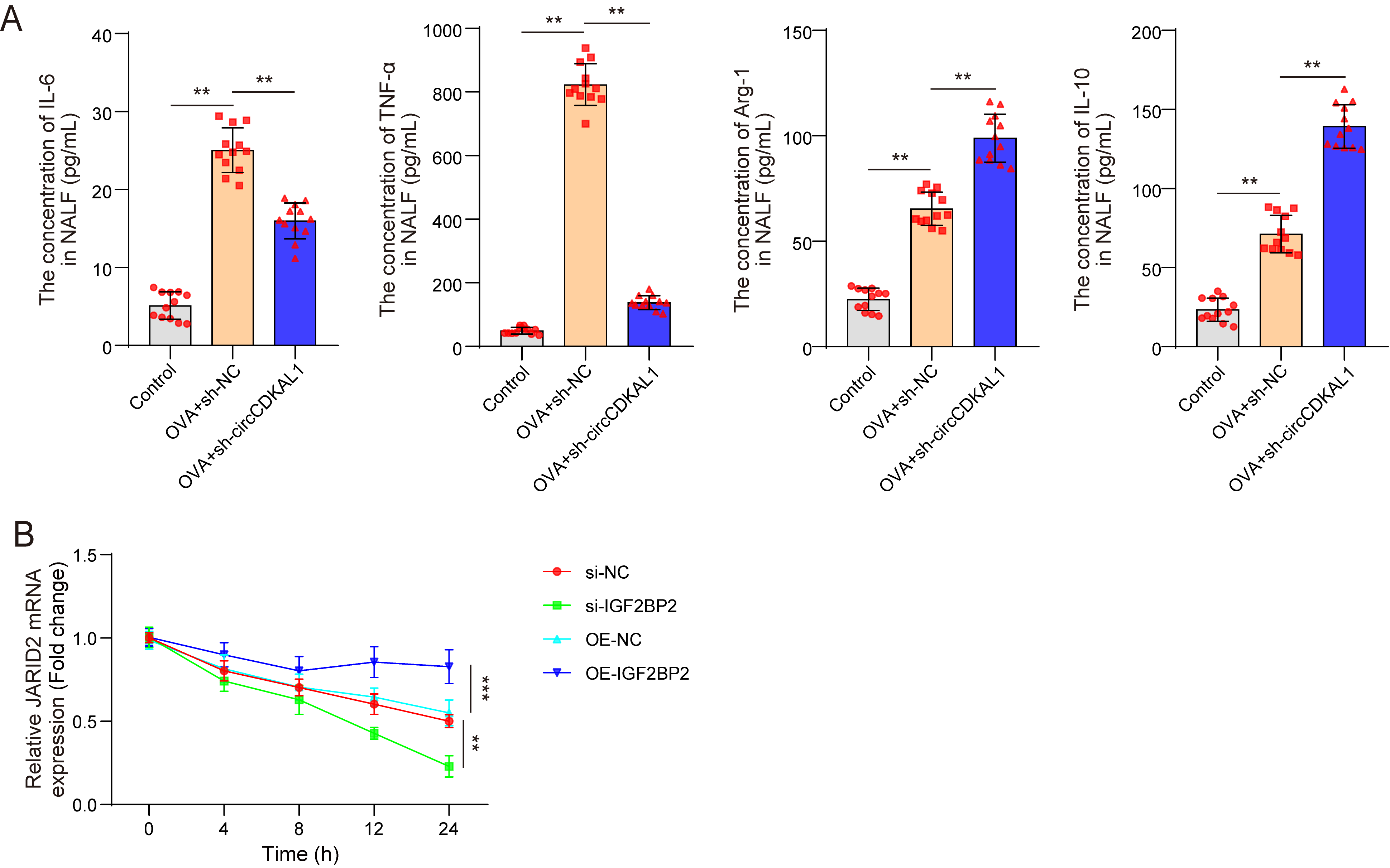

Supplement: Supplementary file 2 — Supplementary Figure 1 [file 41420_2025_2710_MOESM2_ESM.tif]

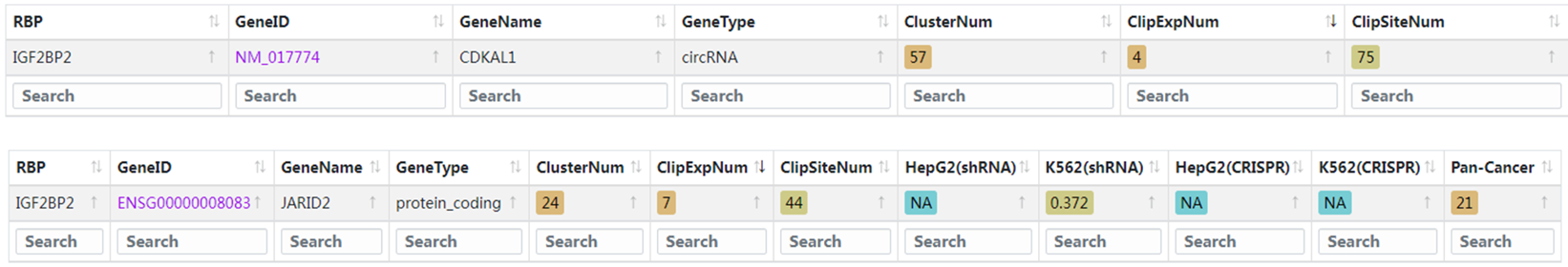

Supplement: Supplementary file 4 — Supplementary File 1 [file 41420_2025_2710_MOESM4_ESM.tif]

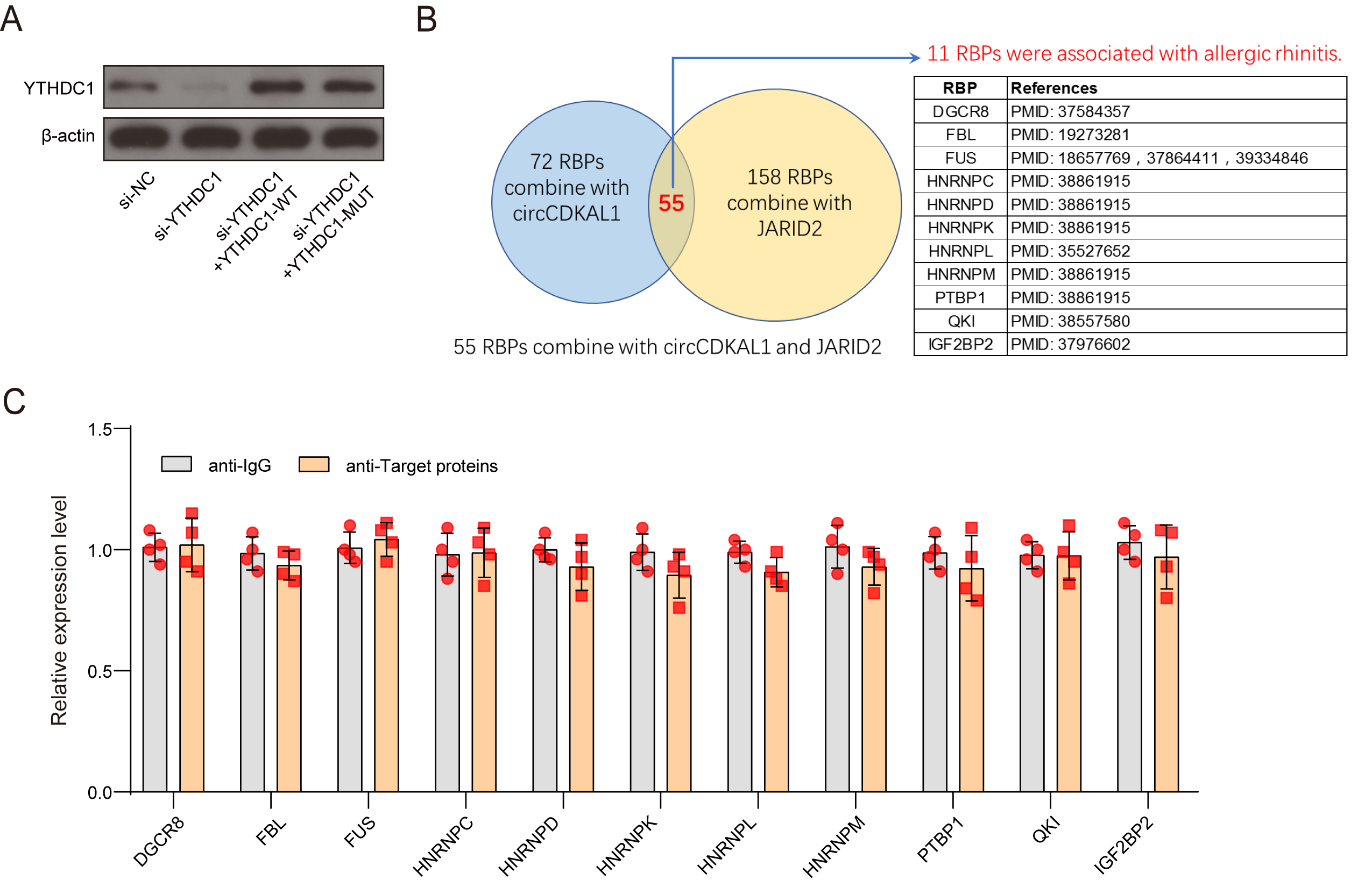

Supplement: Supplementary file 5 — Supplementary File 2 [file 41420_2025_2710_MOESM5_ESM.tif]
