# Supplementary material for: METTL3-mediated m6A modification of circCDKAL1 regulates macrophage M1 polarization and nasal epithelial cell barrier function in allergic rhinitis through IGF2BP2/JARID2/HMGB1 axis
Source: Cell Death Discov. 2025 Aug 29;11:417. doi: 10.1038/s41420-025-02710-7 (PMC12397420; doi:10.1038/s41420-025-02710-7)
Supplement: Supplementary file 3 — Supplementary Figure Legend [file 41420_2025_2710_MOESM3_ESM.docx]

**Supplementary Figure 1 The levels of IL-6, TNF-α, Arg-1and IL-10 in NALF of AR mice and the influence of IGF2BP2 silencing on JARID2 mRNA stability in HNEpCs**

(A) The levels of IL-6, TNF-α, Arg-1 and IL-10 in NALF of AR mice were examined by ELISA. n=12. (B) JARID2 mRNA stability in HNEpCs with sh-IGF2BP2 or OE-IGF2BP2 transfection was detected using RT-qPCR. n=4. ***P*<0.01, ****P*<0.001.
